# Supplementary figures and images for: Modulation of KDM1A with vafidemstat rescues memory deficit and behavioral alterations
Source: PLoS One. 2020 May 29;15(5):e0233468. doi: 10.1371/journal.pone.0233468 (PMC7259601; doi:10.1371/journal.pone.0233468)

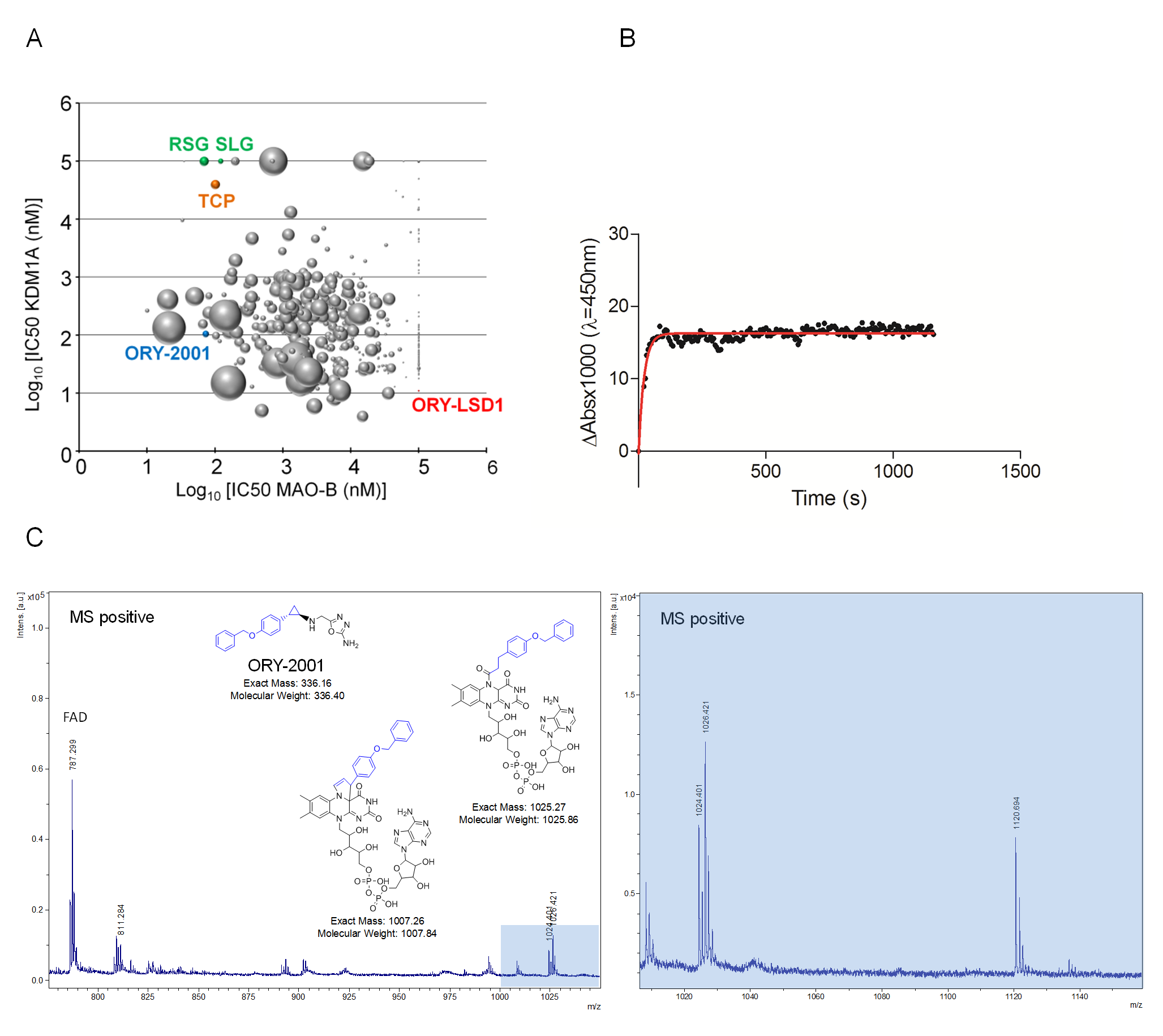

Supplement: S1 Fig — (A) Bubble graph representing KDM1A, MAO-B and MAO-A IC50 values inhibitors. MAO-B and KDM1A are represented on the x and y-axis, and the MAO-A activity is represented as the size of the bubble. The dual KDM1A/MAO-B inhibitor ORY-2001 (blue), selective KDM1A inhibitor ORY-LSD1 (red), MAO-B inhibitors rasagiline(RSG) and selegiline (SLG) (green) and dual MAO-A/B inhibitor tranylcypromine (TCP, orange) have been labeled in the graph. (B) Time course of the change in absorption at 450 nM due to binding of ORY-2001 (40 μM) to the FAD co-factor in KDM1A (15 μM). (C) Maldi-Tof analysis confirms adduct formation between ORY-2001 and KDM1A. (TIF) [file pone.0233468.s001.tif]

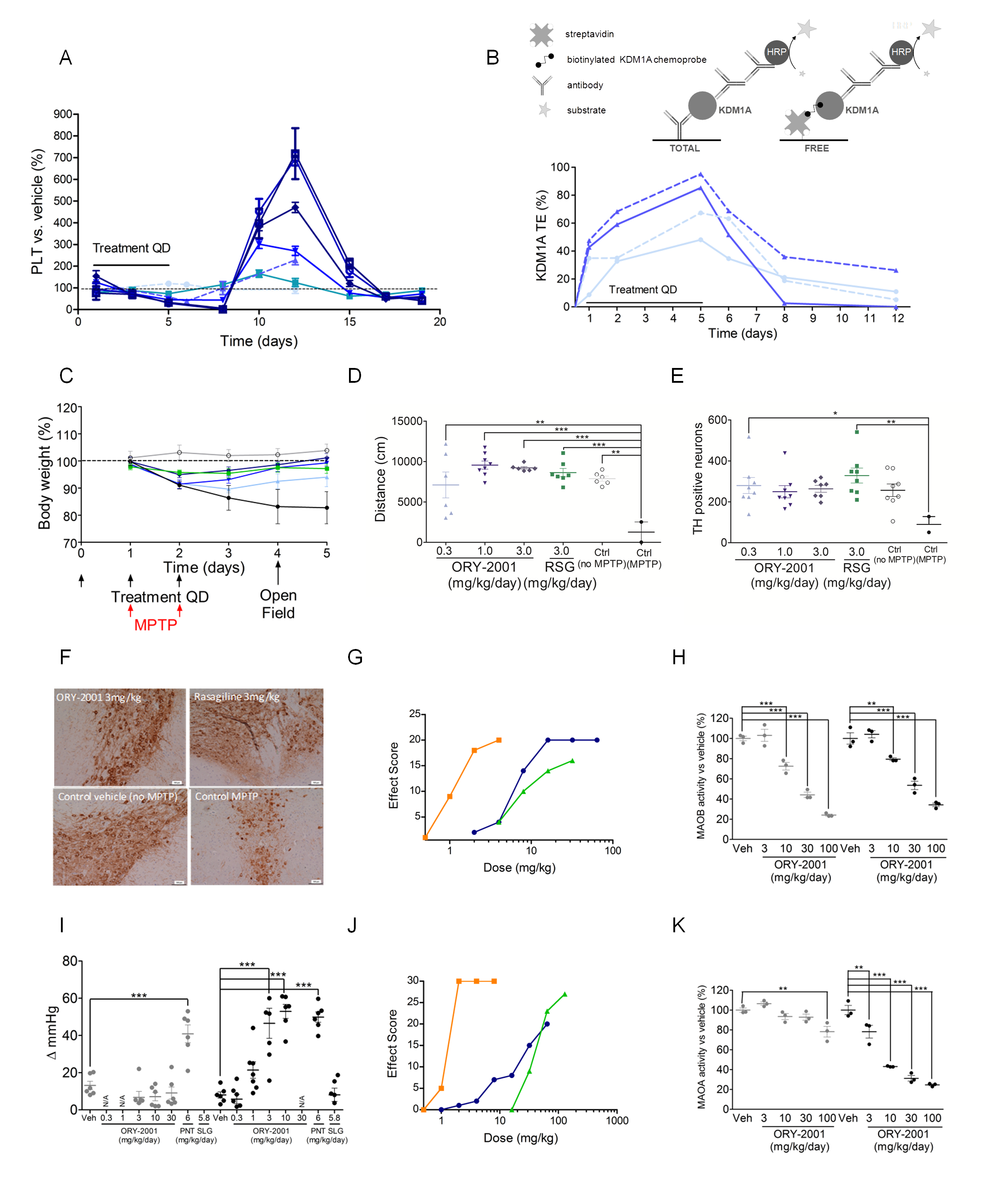

Supplement: S2 Fig — KDM1A inhibition: (A) Dose and time response of platelet levels in male Wistar rats during and after 5 days of treatment with ORY-2001 at ●0.06 mg/kg (N = 3), ■0.2 mg/kg (N = 6), ▲0.4 mg/kg (N = 3), ▼0.6 mg/kg (N = 6), ◆2 mg/kg (N = 6), o 6 mg/kg (N = 6), □ 20 mg/kg (N = 6). (B) KDM1A Target Engagement analysis in rat. Top: schematic representation of the sandwich ELISA assay used for the determination of total and free KDM1A. Bottom: dose- and time-dependent response of KDM1A Target Engagement (%) measured in brain (solid line) and in PBMCs (dotted line) in male Sprague-Dawley rats treated for 5 days with ORY-2001 at ● (light blue) 0.06 mg/kg (N = 3) or ▲ (dark blue) 0.4 mg/kg (N = 3). MAO-B inhibition: Preventive effect of i.p. administration of ORY-2001 in an MPTP induced Parkinsonism model in C57BL/6 mice on (C) body weight: ORY-2001 (blue) at ▲ 0.3 mg/kg ▼ 1 mg/kg ◆3.0 mg/kg; RSG (green) at ■ 3 mg/kg, with ○ Control without MPTP, or ● Control vehicle + MPTP; (D) locomotor effect- horizontal distance; (E) TH+ neurons (N = 8/group); (F) representative image of histopathology. (G) Potentiation of PEA-induced symptoms in CD1 mice treated with ● ORY-2001 (blue), ED50 = 6.4 mg/kg, ■ TCP (orange), ED50 = 1.1 mg/kg, ▲ SLG (green), ED50 = 8.0 mg/kg; cumulative score from N = 10/group. (H) Ex vivo measurement of MAO-B target inhibition in CD1 mice. Gray dot (acute), Black dot (chronic 5 days). MAO-A inhibition: (I) Tyramine pressor response in Wistar rats. Gray dot (acute), black dot (chronic 5 days); N = 6-7/group. (J) Potentiation of L-5-HTP-induced symptoms in C57BL/6 mice after treatment with ● ORY-2001 (blue), ■ TCP (orange), ▲ SLG (green); cumulative score from N = 10/group; (K) Ex vivo measurement MAO-A target inhibition in the brain of CD1 mice. Gray dot (acute), black dot (chronic 5 days). All treatments were QD per oral gavage unless stated otherwise. Means ± SEM are represented. Different drug treatments were compared by Oneway-ANOVA with Dunnett analysis. *p [file pone.0233468.s002.tif]

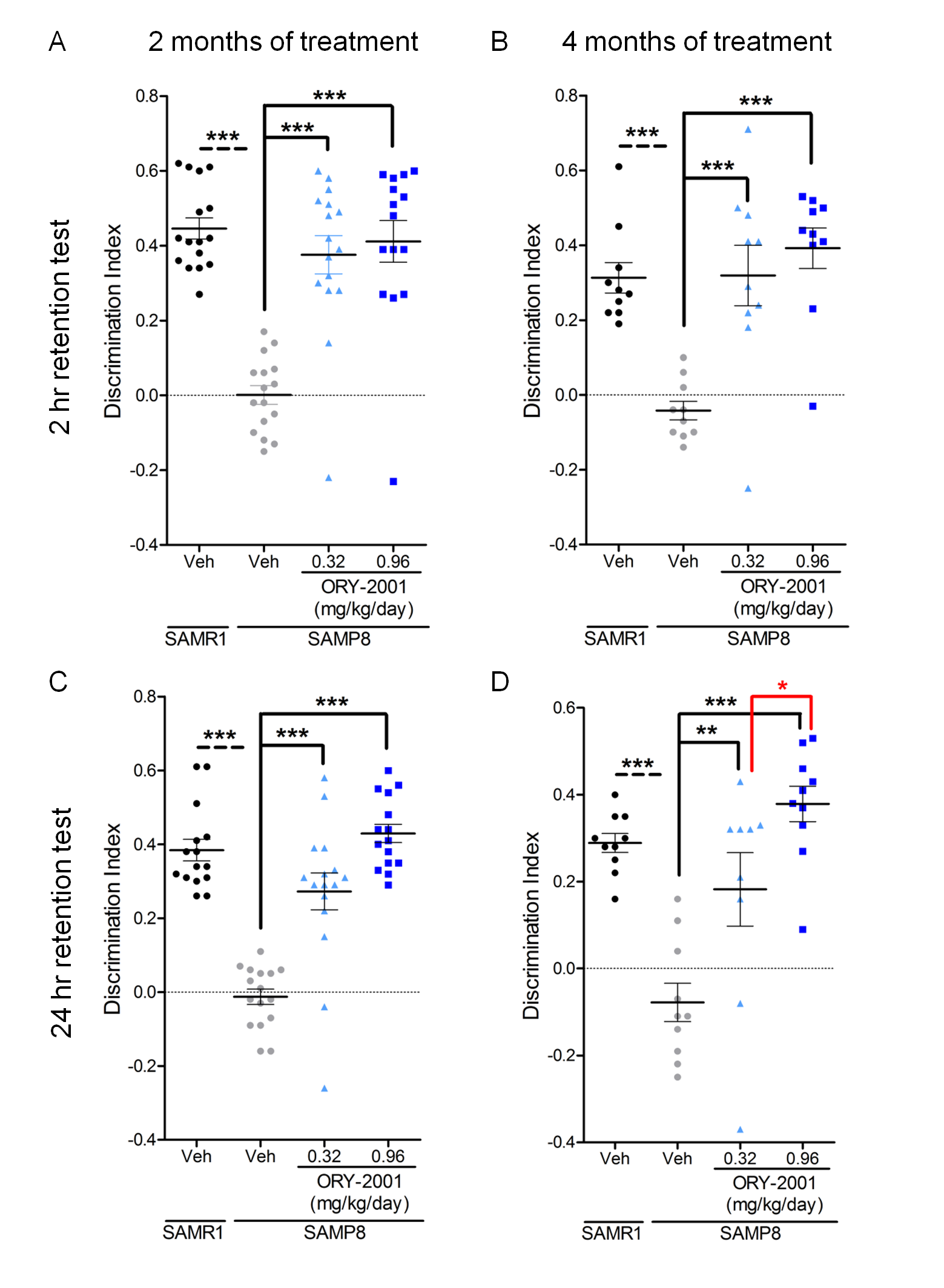

Supplement: S3 Fig — ORY-2001 treatment effect on the DI in the NORT in male SAMP8 mice. The retention test was evaluated 2 (A,B) and 24 (C,D) hours after training to measure changes in medium and long term memory. Five month old animals were divided in two groups receiving 2 (N = 15-16/group) (A,C) and 4 (N = 9-10/group) (B,D) months of treatment. Means and SEM are represented. SAMR1 and SAMP8 vehicle groups were compared by t-Test. Among the SAMP8 cohorts, different drug treatments were compared by oneway-ANOVA with Dunnett and SNK post-Hoc analysis. **p < 0.01, ***p < 0.001. (TIF) [file pone.0233468.s003.tif]

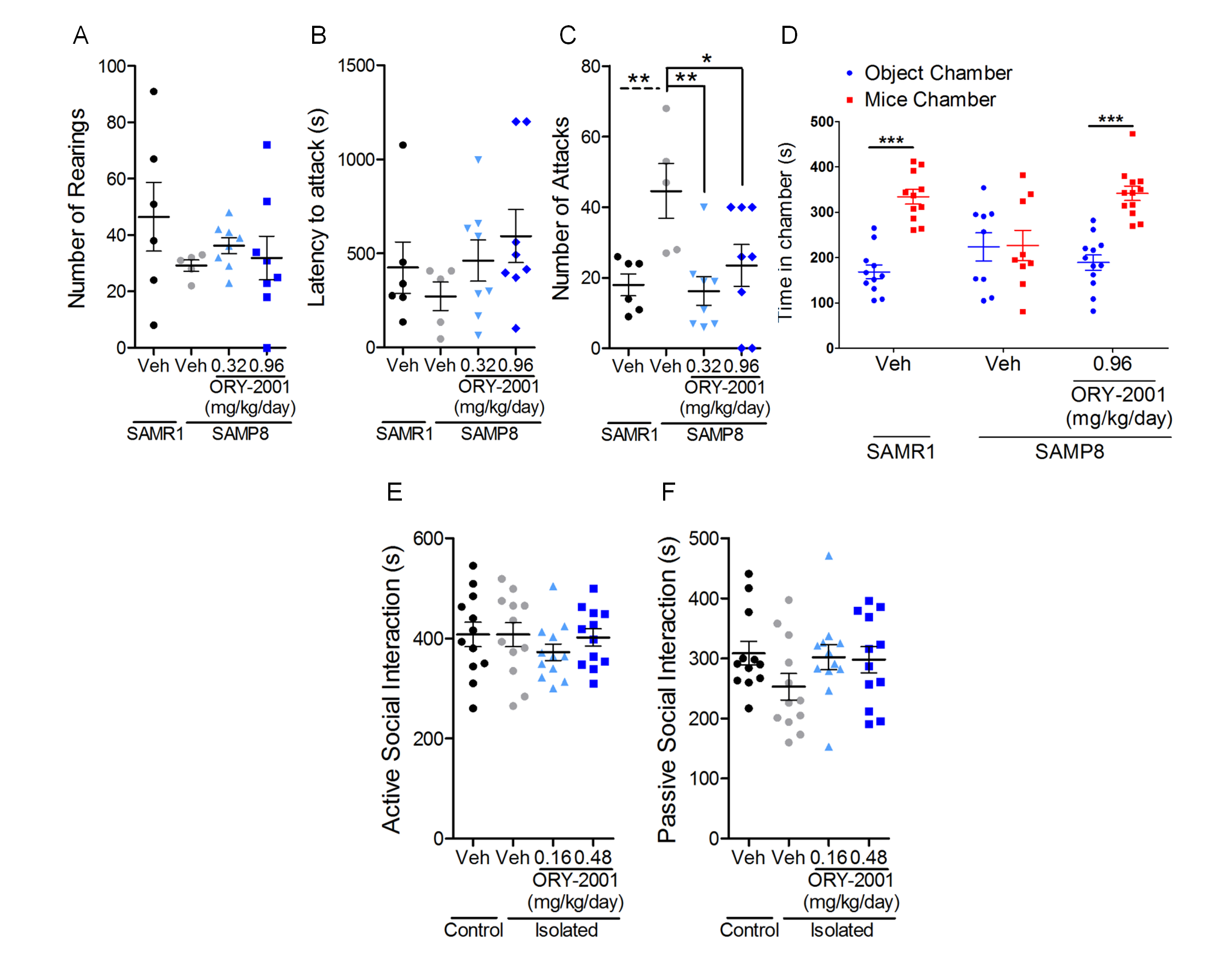

Supplement: S4 Fig — Social behavior in SAMP8 mice: (A) number of rearings in the RI test performed, (B) latency to attack and (C) number of attacks of vehicle treated SAMR1 and vehicle or ORY-2001 treated SAMP8 male mice (N = 5-8/group). SAMP8 animals did not show significant differences in the number of rearings or latency to attack compared to SAMR1 mice but SAMP8 animals did show higher number of attacks. Treatment with ORY-2001 had no significant effect on the number of rearings in SAMP8, but a dose dependent tendency to increase in the latency to attack was observed and a clear effect to reduce the number of attacks. (D) In the Three Chamber Test, SAMP8 animals do not show preference for the chamber with the novel mice, treatment with ORY-2001 restored the normal preference to similar levels observed in the SAMR1 (N = 9-12/group). SAMR1 and SAMP8 were compared by t-TEST. Means and SEM are represented. Vehicle and drug vs vehicle treatments in SAMP8 mice or isolated rats were compared by oneway-ANOVA with Dunnett and SNK post-Hoc analysis. Means and SEM are represented. *p < 0.05, **p < 0.01, ***p < 0.001. Social behavior in the rat isolation rearing model: Time spent on (E) active and (F) passive social interactions in the RI test performed on vehicle treated control and vehicle or ORY-2001 treated isolated rats (N = 12/group). Isolated rats did not show significant differences in active or passive social interaction compared to non isolated rats and treatment with ORY-2001 had no effect on these parameters. Control and Isolated vehicle groups were compared by t-Test. Means and SEM are represented. Vehicle and drug vs vehicle treatments in SAMP8 mice or isolated rats were compared by oneway-ANOVA with Dunnett and SNK post-Hoc analysis. *p < 0.05, **p < 0.01, ***p < 0.001. (TIF) [file pone.0233468.s004.tif]

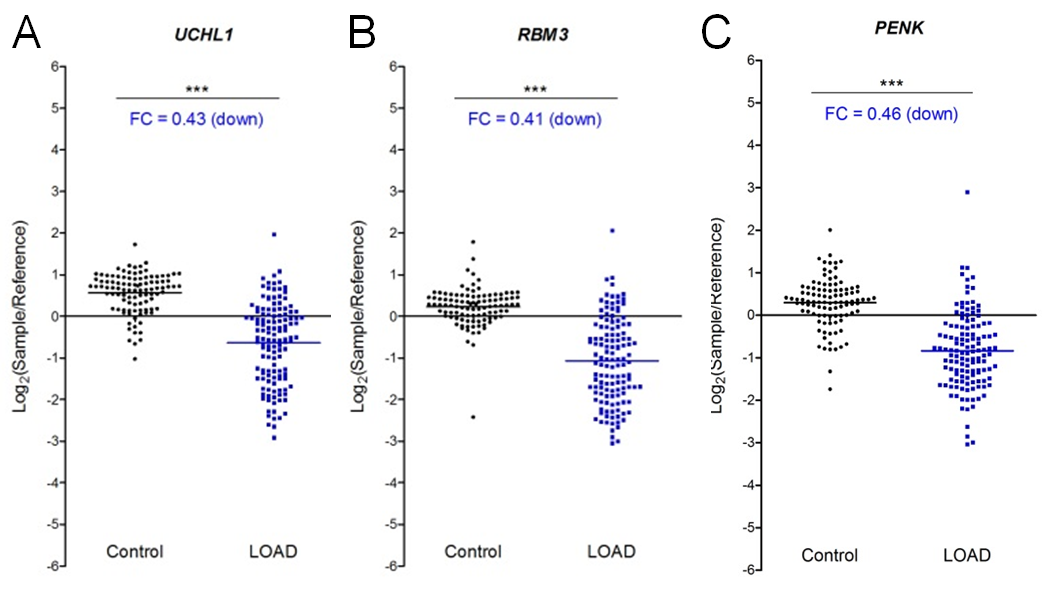

Supplement: S5 Fig — Re-examination of the expression of the orthologues of SAMP8 biomarkers shows differential expression of synaptic plasticity genes (E) UCHL1 (F) RBM3, (G) PENK in human prefrontal cortex of Control and LOAD samples from NCBI GEO GSE44770 [44]. All samples are represented as Log2 (sample/reference sample). Control: N = 101 and LOAD: N = 129 subjects. Means ± SD are represented. Fold changes (FC) were calculated as 2^[average Log2 (LOAD/reference values)–average Log2 (Control/reference values)]. Significance was calculated by Mann-Whitney test. ***p < 0.001. (TIF) [file pone.0233468.s005.tif]

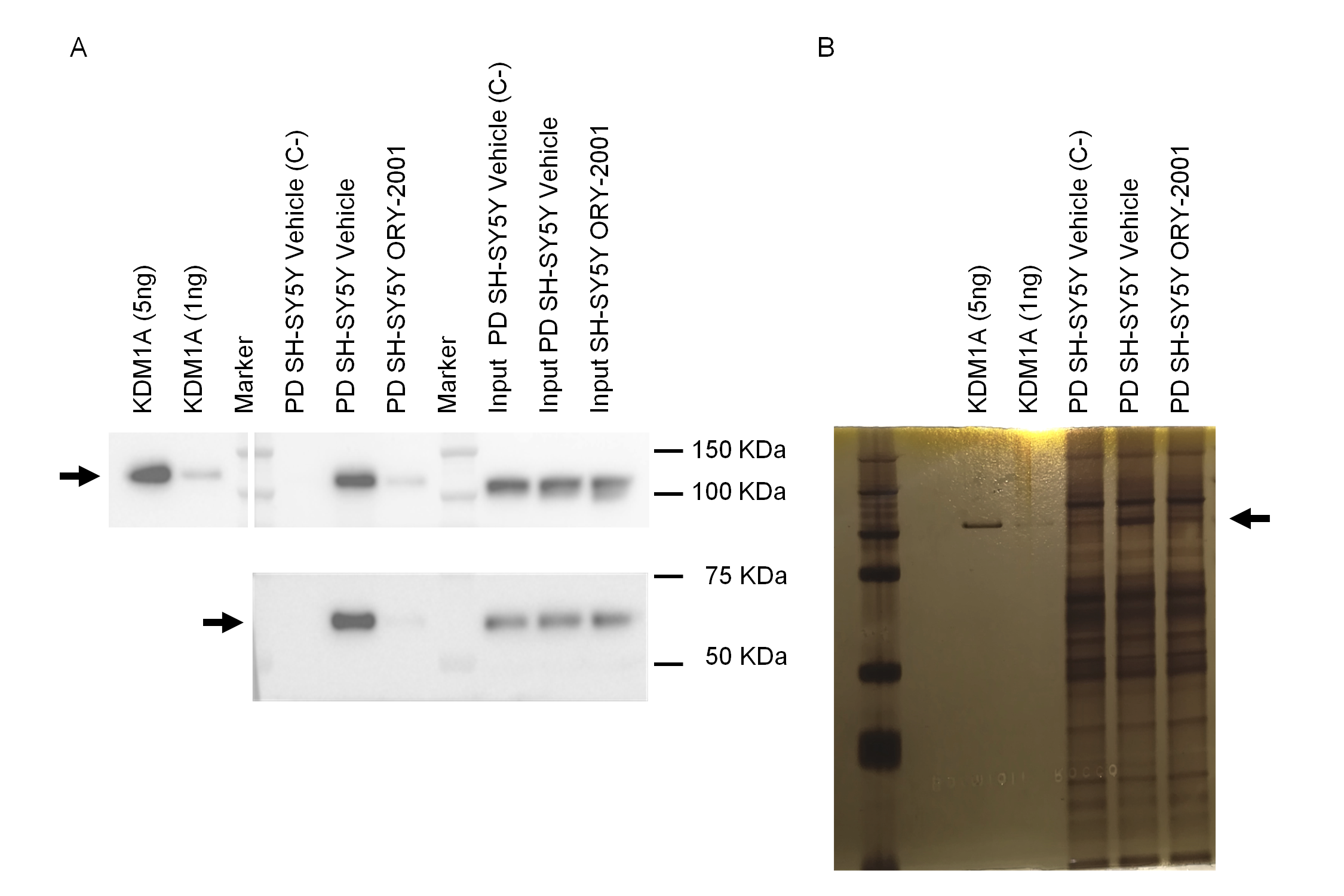

Supplement: S6 Fig — (A) Western blot of recombinant KDM1A and vehicle or ORY-2001 treated SH-SY5Y input and KDM1A chemoprobe pulldown (PD) samples, analyzed with anti-KDM1A (top) and anti-RCOR1 (bottom) antibodies. (B) Silver nitrate staining of recombinant KDM1A and vehicle or ORY-2001 treated SH-SY5Y input and KDM1A chemoprobe pulldown (PD) samples analysed by PAGE. C-: negative control (pulldown of vehicle treated cells in absence of chemoprobe). 10% of the total pulldown was loaded per lane. (TIF) [file pone.0233468.s006.tif]
