# Supplementary material for: Modulation of KDM1A with vafidemstat rescues memory deficit and behavioral alterations
Source: PLoS One. 2020 May 29;15(5):e0233468. doi: 10.1371/journal.pone.0233468 (PMC7259601; doi:10.1371/journal.pone.0233468)
Supplement: S1 File — (DOCX) [file pone.0233468.s014.docx]

S1 File. Supporting Data

## Synthesis of ORY-2001

The synthetic process used for the preparation of ORY-2001 [1] involves two stages starting from (1R,2S)-2-(4-(benzyloxy)phenyl)cyclopropan-1-amine (source) and tert-butyl (5-(chloromethyl)-1,3,4-oxadiazol-2-yl)carbamate.

Step 1

Tert-butyl (5-(chloromethyl)-1,3,4-oxadiazol-2-yl)carbamate (141 mg, 0.606 mmol) was added to a solution of commercially available (1R,2S)-2-(4-(benzyloxy)phenyl)cyclopropan-1-amine (145 mg, 0.606 mmol) and K_2_CO_3_ (166 mg, 1.213 mmol) in dry DMF (1.5 mL) and stirred at RT for 2 hr. After completion, the reaction mixture was poured into ice water (10 mL) an extracted with EtOAC (4 x 10 mL). The combined organic extracts were washed with water (3 x 10 mL), brine (10 mL), dried over anhydrous Na_2_SO_4_, filtered and concentrated under vacuum. The residue obtained was purified by column chromatography (SiO_2_) using MeOH:CHCl_3_ (1:99) as eluent to afford tert-butyl (5-((((1R,2S)-2-(4-(benzyloxy)phenyl)cyclopropyl)amino)methyl)-1,3,4-oxadiazol-2-yl)carbamate (100 mg, 37.7 %) as a pale green liquid.

Step 2

To a solution of tert-butyl (5-((((1R,2S)-2-(4-(benzyloxy)phenyl)cyclopropyl)amino)methyl)-1,3,4-oxadiazol-2-yl)carbamate (100 mg, 0.229 mmol) in 1,4-dioxane (1 mL) at 0ºC was added HCl in dioxane (1 mL) and stirred for 18 hr. After completion, the solvent was evaporated and the residue was dissolved in water (10 mL), basified with Na_2_CO_3_ solution, extracted with EtOAc (3 x 5 mL). The combined extracts were washed with water (5 mL), brine (5 mL), dried over anhydrous Na_2_SO_4_, filtered and evaporated The crude residue was purified by column chromatography (SiO_2_) using MeOH:CHCl_3_ (5:95) as eluent to afford 5-((((1R,2S)-2-(4-(benzyloxy)phenyl)cyclopropyl)amino)methyl)-1,3,4-oxadiazol-2-amine (40 mg, 52 %) as a white solid.

1H-NMR (400 MHz, DMSO-d6, ppm): 0.787 to 0.866 (2H, m), 1.673 to 1.719 (1H, m), 2.159 (1H, m), 2.960 (1H, s), 3.711 to 3.723 (2H, d), 5.012 (2H, s), 6.816 to 6.848 (4H, m), 6.892 to 6.914 (2H, m), 7.295 to 7.431 (5H, m). Mass (M+H): 337.1

HPLC Purity: HPLC analysis was performed using an Acquity UPLC BEH C18 (100mm X 2.1mm, 1.7μm) column with mobile phase: 0.025 % TFA in a gradient H_2_O/CAN with gradient: time/ % B: 0/30, 4/80, 6/80, 6.1/30 at a flow rate 0.4 mL/ min at room temperature. UV analysis was at 229 nm and ORY-2001 t_R_ = 1.64 min. Several batches with purity > 96 % were synthesized, most experiments were run using batch 7 with purity of 98.40%.

## Pharmacokinetics, Pharmacodynamics and *in vivo* Pharmacological Activity and Selectivity

The pharmacokinetics, bioavailability and brain-to-plasma ratios were assessed after intravenous (i.v.) (2 mg/kg) or oral (p.o.) (10 mg/kg) administration of ORY-2001 to male Balb/c mice. ORY-2001 displayed an adequate half-life (T_1/2_ = 133 min) and high exposure as represented by elevated maximum concentration (Cmax = 4,159 ng/ml) and area-under-the-curve (AUC = 1,031,580 min•ng/mL) values after p.o. administration, as well as excellent oral bioavailability (F = 75%) and good brain penetration (brain to plasma AUC_last_ = 1.4).

The pharmacokinetics, bioavailability and brain-to-plasma ratios were also assessed after i.v. (2 mg/kg) or p.o. (10 mg/kg) administration of ORY-2001 to male Wistar rats. ORY-2001 displayed an adequate half-life (T_1/2_ = 197 min) and plasma C_max_ = 3,398 ng/ml and area-under-the-curve (AUC = 1,259,580 min•ng/mL) values after oral administration, as well as excellent oral bioavailability (F=95%) and good brain penetration (brain to plasma AUC_last_ = 1.5).

Finally, we also evaluated the PK of ORY-2001 administered in drinking water. The C_max_ and AUC for administration by oral gavage was 3.5 and 1.6 fold higher than for continuous administration in drinking water. A summary of the C_max_ and AUC dose equivalencies for the different treatment schemes in rats and mice is represented in Table S2a.

## Pharmacology of ORY-2001: KDM1A inhibition

KDM1A is involved in hematopoiesis, and it is well known that KDM1A knockdown or KDM1A inhibitors can halt hematopoiesis when administered at sufficiently high doses and during sufficient time^(39)^. The impact and reversibility of the effect of ORY-2001 (0.02-20 mg/kg p.o. gavage) on hematology was assessed during a 5 day treatment and 14 day recuperation period in rats. A dose dependent reduction in hematological parameters was observed, exemplified in Fig. S2a by the evolution of platelet (PLT) levels. The PLT nadir was reached between day 5 and 8, and maximum rebound was observed around day 12. The maximum effect as assessed by the magnitude of the rebound was reached at 6 mg/kg, the EC_50_ of PLT reduction was ~ 0.5 mg/kg. Data from N = 6 rats are represented as mean ± SEM.

A chemoprobe-based immune assay (Mascaró *et al*., manuscript submitted) was used to assess KDM1A target engagement dynamics of ORY-2001 administered at 0.06 and 0.4 mg/kg p.o. gavage in rat PBMC and brain samples (Fig S2b). Dose dependent target engagement was observed in PBMCs and brain samples, which reached a maximum after 5 days of administration and showed an estimated half-life of 3 days. These data confirm that ORY-2001 crosses the BBB and inhibits brain KDM1A.

## Pharmacology of ORY-2001: MAO inhibition

MAO-A and MAO-B are key enzymes that degrade biogenic and dietary amines, yet the proteins display distinct substrate selectivity: MAO-A preferentially oxidizes 5-HT or NE, and MAO-B preferentially oxidizes β-phenylethylamine (PEA).

The capacity of ORY-2001 to inhibit brain MAO-B was tested in the mouse 1-methyl-4-phenyl-1,2,3,6-tetrahydropyridine (MPTP) model. MPTP is a pro-toxin that is converted to the highly neurotoxic cation 1-methyl-4-phenylpyridinium (MPP+) by MAO-B. MPP+ primarily kills dopamine-producing neurons in the pars compacta of the substantia nigra and causes permanent symptoms of PD (or even death) by destroying dopaminergic neurons. MAO-B knockout mice are resistant to MPTP and MAO-B inhibitors protect mice from its toxicity.

Animals were administered 0.3 to 30 mg/kg of ORY-2001 i.p. to see if this could prevent MPTP neurotoxicity. RSG, a selective MAO-B inhibitor, was used as a positive control. The MPTP insult provoked weight loss and strong locomotor effects in 2/10 vehicle treated animals, and killed the remaining ones. RSG administered i.p. at 3 mg/kg and ORY-2001 at doses between 1 and 30 mg/kg provided full protection against the MPTP insult; and ORY-2001 at 0.3 mg/kg provided partial protection (shown for doses of Fig. S2c-f). MAO-B inhibition was also assessed in the PEA model. CD1 mice were administered ORY-2001, TCP or SLG p.o. gavage, followed by 25 mg/kg PEA, and PEA induced symptoms were evaluated. The dose curve of the effect scores for ORY-2001, TCP and SLG showed an ED_50_ of 6.4, 1.1 and 8.0 mg/kg, respectively (Fig S2g). Finally, brain MAO-B activity was measured *ex vivo* after acute or subchronic administration of ORY-2001 (p.o. gavage) to CD1 mice. Acute and chronic treatment with ORY-2001 yielded an EC_50_ around 20 mg/kg ORY-2001 (Fig S2h). The differences in *in vivo* MAO-B potency in the MPTP and the PEA based assays could reflect pharmacokinetics differences due to route of administration, but also differences in assays, substrate selectivity or site of action evaluated. These data show that ORY-2001 is a more potent inhibitor of KDM1A than of MAOB *in vivo* .

The irreversible MAO-A inhibitor TCP was shown to be effective to treat major depression, yet its clinical use has dropped sharply due to notorious on-target food and drug interactions. MAO-A is key to the metabolism of tyramine in food and when intestinal and hepatic MAO-A are inhibited, the “first-pass” clearance of tyramine is blocked and tyramine levels can rise and provoke hypertensive crises that can result in stroke or cardiac arrhythmia.

*In vitro*, ORY-2001 exhibits a 50-fold selectivity window for MAO-B over MAO-A. To assess the impact of ORY-2001 on MAO-A *in vivo*, we investigated the effects of the interaction of ORY-2001 with the tyramine pressor response in male Sprague Dawley (SD) rats after acute and subchronic dosing by oral gavage. No tyramine response was detected with ORY-2001 administered at acute doses up to 30 mg/kg. Sub-chronic dosing of ORY-2001 did not lead to a significant increase in tyramine-induced pressor response in rats at doses up to 1 mg/kg (Fig S2i). The MAO-A inhibitory activity was also evaluated in the (L-5-HTP) model. CD1 mice were administered ORY-2001, TCP or SLG p.o. gavage, followed by 100 mg/kg L-5-HTP i.p., and the induced symptoms were evaluated. The dose curve of the effect scores for ORY-2001, TCP and SLG showed an ED_50_ of 20.5, 1.4 and 42.2 mg/kg (Fig S2j), respectively; yielding a relative MAO-B (PEA) to MAO-A (L-5-HTP) activity of 3.2, 1.3 and 5.2 fold for the 3 compounds under the test conditions. MAO-A activity was also measured in mice brain after acute or 5 d sub-chronic administration of 1 to 100 mg/kg ORY-2001 (p.o. gavage) in an ex vivo assay. In the acute setting, only the 100 mg/kg dose led to a significant inhibition of MAO-A. Sub-chronic dosing of ORY-2001 significantly inhibited MAO-A with an ED_50_ around 10 mg/kg; (Fig S2k), i.e at 20 times higher concentration than the EC_50_ for platelet reduction in the same species treated in the same manner.

In summary, we demonstrated that in rodents *in vivo* ORY-2001 inhibits KDM1A > MAO-B > MAO-A; and that the dose limiting toxicity of ORY-2001 would be expected to be delimited by the hematopoietic impact.

## References

1. Ortega Muñoz A, Fyfe MCT, Martinell Pedemonte M, Tirapu Fernandez De La Cuesta, I, and Estiarte-Martínez M, inventors; Oryzon Genomics S.A., assignee. Arylcyclopropylamine based demethylase inhibitors of LSD1 and their medical use. World Intellectual Property Organization WO2012/013728. filed July 29, 2010, published February 2, 2012.
