# Supplementary material for: Modulation of KDM1A with vafidemstat rescues memory deficit and behavioral alterations
Source: PLoS One. 2020 May 29;15(5):e0233468. doi: 10.1371/journal.pone.0233468 (PMC7259601; doi:10.1371/journal.pone.0233468)
Supplement: S2 File — (DOCX) [file pone.0233468.s015.docx]

# S2 File: Supporting Materials and Methods

## Contact for reagents and resource sharing

Further information and requests for reagents should be directed to and will be fulfilled by the communicating author Tamara Maes ([tmaes@oryzon.com](mailto:tmaes@oryzon.com)).

## Experimental models and samples

### Cell lines

Human leukemic THP1 (ACC-16) cells (male) [1] were obtained from DMSZ and maintained in RPMI 1640 medium (Cat# R0883; Sigma Aldrich) supplemented with 10 % heat inactivated fetal bovine serum (FBS; Cat# F9665; Sigma-Aldrich) and 2 mM Glutamine (Cat# X0550 Biowest).

Human neuroblastoma SH-SY5Y (Cat# 93030304) cells were obtained from Sigma-Aldrich and maintained in 1:1 Ham’s F12 (cat.# N4888, Sigma-Aldrich):MEM (Cat.# M2279, Sigma-Aldrich), supplemented with 15% heat inactivated foetal bovine serum (Cat.# F9665, Sigma-Aldrich), 2mM Glutamine (Cat.# GLN-B, Labclinics) and 1% Non Essential Amino Acids NEAA (Cat.# M7145, Sigma-Aldrich).

Cells were maintained in a 5%CO_2_ -controlled atmosphere at 37°C in a Thermo Scientific / Forma Series II Model 3100 cell culture incubator.

### Human brain samples

Post-mortem human brain samples for histology used included Familial and Sporadic Alzheimer’s Disease (AD) etiology in different clinical stages of the pathology (healthy aged control n = 7, 1 female and 6 males, average age 49,4; AD sporadic stage I-II/C n = 9, 2 females and 7 males, average age 66,3; AD sporadic stage III-IV/C n = 7, 3 females and 4 males, average age 78,3; AD sporadic stage V-VI/B n = 8, 5 females and 3 males, average age 77,5). Frontal Cortex and Hippocampus were analyzed. Brain tissue was obtained from the HUB-ICO-IDIBELL Biobank following the informed written consent and according to Spanish legislation (Real Decreto de Biobancos 1716/2011) and approval of the local ethics committees (Comité Ético de Investigación Clínica (CEIC) del Hospital Universitario de Bellvitge).

### Human CSF samples

Human CSF samples from patients with Alzheimer’s disease and controls (1 ml CSF/donor) were sourced from Precision Medicine, Solana Beach, U.S.A. Selected Alzheimer’s donors, obtained in compliance with legislation (Protocol No 8009, Western Institutional Review Board) and Selected control donors (Protocol No 7005/7009, Western Institutional Review Board). All subjects signed approved written informed consent prior to any entry procedures being performed.

### Mouse pharmacokinetics

To assess the PK and bioavailability and brain/plasma ratios of oral and iv administered ORY-2001; the compound was administered by intravenous bolus (2 mg/kg in vehicle 10 % DMSO in 20 % aq. HPβCD) or by oral gavage (10 mg/kg in vehicle 20 % aq. HPβCD) to 8-10 week old male Balb/c mice (N = 3 x 3 each for the IV and PO group) of 25-30 g weight, procured from Raj Biotech, Pune, India. Temperature and humidity were maintained at 22 ± 3 ºC and 40-70 %, respectively and illumination was controlled to give a sequence of 12 hr light / dark cycle. The temperature and humidity were recorded by auto-controlled data logger system. All the animals were acclimatized to the experimental conditions for at least three days prior to dosing. All the animals were provided laboratory rodent diet (Vetcare India Pvt. Ltd, Bengaluru) except for overnight before treatment and 2 hr post dose. Reverse osmosis water treated with ultraviolet light was provided *ad libitum*. Blood samples were collected at 0, 0.05, 0.16, 0.5, 1, 2, 4, 8 & 24 hr for IV and for PO at 0, 0.25, 0.5, 1, 2, 4, 6, 8 and 24 hr post dosing. Each mouse was assigned to one of 3 subgroups and sampled at 3 timepoints. Animals were euthanized by using excess CO_2_ asphyxiation. The study was conducted at Sai Life Sciences Limited, Pune, India, in accordance with the guidelines of the Institutional Animal Ethics Committee (IAEC) and in accordance with the requirement of Committee for the Purpose of Control and Supervision of Experiments on Animals (CPCSEA), India. Prior approval was obtained before initiation of the study (IAEC Protocol No. IAEC/PRT/012-10; IAEC-CPCSEA).

### Rat pharmacokinetics

To assess the PK, bioavailability, brain/plasma ratios of oral and iv administered ORY-2001; the compound was administered by intravenous bolus (2 mg/kg in vehicle 10 % DMSO in 20 % aq. HPβCD) or oral gavage (3, 10, 30, 60 and 100 mg/kg in 2% Tween 80 and 20 % aq. HPβCD) to male Wistar rats (N = 4 per dose level) or oral gavage (10 mg/kg in 2% Tween 80 and 20 % aq. HPβCD, N = 9) of 10-12 weeks of age, procured from Raj Biotech, Pune, India were housed under the same conditions as in the previous section. Blood samples were collected at 0, 0.08 (for IV only), 0.25, 0.5, 1, 2, 4, and 6 (Only for PO) 8 and 24 hr for IV and PO group and at three rats were euthanized by using excess CO_2_ asphyxiation, each at 0.25, 1 and 4 hr post dose and brain was collected. The study was conducted at Sai Advantium Pharma Limited, Pune, India, in accordance with the guidelines of the Institutional Animal Ethics Committee (IAEC). Prior approval was obtained before initiation of the study (IAEC Protocol No. IAEC/PRT/006-11).

To assess the PK of ORY-2001 following dose administration by oral gavage versus drinking water in male Wistar rats; ORY-2001 was administered as a single oral dose by oral gavage at 10 mg/kg in 2% Tween 80 and 98% Cyclodextrin 20 % w/v or as oral solution at 0.25 mg/mL in drinking water provided *ad libitum* to male Wistar rats of 8-12 weeks of age, procured from In-vivo Bioscience, India; housed under the same conditions as described in the previous section. Blood samples were collected from each rat at 0.5, 1, 2, 4, 6, 8, 12, and 24 hr (oral gavage) or at 2, 4, 6, 8, 12, 24, 25, 27, 30 and 48 hr (drinking water) were collected from the retro-orbital plexus into labeled tubes, containing K2EDTA solution, as an anticoagulant. Animals were euthanized by using excess CO_2_ asphyxiation. This study was conducted at Sai Advantium Pharma Limited, Pune, India performed as per protocol approved by the Institutional Animal Ethics Committee (IAEC) and in accordance with the requirement of Committee for the Purpose of Control and Supervision of Experiments on Animal (CPCSEA), India. Prior approval was obtained before initiation of the study (IAEC Protocol No. FB-14-069-R-089; IAEC-CPCSEA).

Rat pharmacodynamics

Male Wistar rats (N = 3 or 6 per dose level) of 10-12 weeks of age were maintained in air and temperature controlled cages with regular supply of water and food. A maximum of 3 mice/cage were raised. Before first administration, the animals were marked on the tail for identification. The animals were administered with ORY-2001 for five consecutive days and were maintained two additional weeks for recovery and blood analytics follow up. Animal of each treatment group were sampled for blood on days 1, 3, 5, 8, 10, 12, 15, 17 and 19. Blood samples of days 1, 3 and 5 were obtained just one hr after the compound administration. Blood samples were obtained from leg saphenous vein using recipients treated with EDTA to avoid coagulation. The last day of the study, rats were euthanized under CO2 saturated atmosphere and blood was extracted by cardiac puncture and collected in recipients containing EDTA. The experimental protocol was carried out by Leitat and approved by the Ethics Committee at the Universitat de Barcelona and the Generalitat de Catalunya (ECUB), and it was carried out in accordance to the European Council Directive (2010/63/UE) and Spanish legislation (RD 53/2013). Prior approval was obtained before initiation of the study (Protocol No. 9719; ECUB-UB).

### MPTP neurotoxicity model

Male C57BL/6 mice (8 weeks, 25-30 g) procured from Harlan Interfauna Iberica were housed at PRAAL-PCB in SPF (Specific Pathogen Free) conditions. The temperature range was 18-22 ºC with a 30-70 % relative humidity range. The light / dark cycle was maintained at 12:12 hr (lighting switch on at 7:00 a.m.). All the animals housed at PRAAL-PCB were maintained with 2914 Irradiated Teklad Global 14% Protein Rodent Maintenance Diet (Harlan) provided *ad libitum*. Autoclaved tap water was provided *ad libitum*. The animals were randomly divided into groups (each N = 8) and treated on day 0, day 1, and day 2 with ORY-2001 (0.3, 1, 3, 10 or 30 mg/kg, i.p.), with RSG (3 mg/kg, i.p.) as positive control, or with vehicle as positive or blank control. On day 1 and 2, one hr after the administration, all animals except the blank group were injected a dose of MPTP (40 mg/kg, s.c.). 48 hr later, animals were tested for locomotor activity.

The health and welfare assessment of this study was carried out according to an observation protocol monitoring: body weight, general condition/body condition, reactivity (with the environment) and motor activity, petechiae and haemorrhages, dehydration and survival. After final observation assessment, animals were euthanized with CO2 atmosphere, and brains collected for immunohistochemistry in the SNc (substancia nigra compacta) of control and treated animals. Studies were performed in accordance with the institutional guidelines for the care and use of laboratory animals (European Communities Council Directive 86/609/EEC) established by the Ethical Committee for Animal Experimentation (ECAE) at the PRAAL-PCB. Prior approval was obtained before initiation of the study (Protocol No. 5020; ECAE-PRAAL-PCB).

### Mouse PEA behavior model

Male C57BL/6 mice (8 weeks of age, 25-30 g) procured from Harlan Interfauna Iberica were housed at animal facility of Parc Cientific de Barcelona. The temperature range was 18-22 ºC with a 30-70 % relative humidity range. The light / dark cycle was maintained at 12:12 hr (lighting switch on at 7:00 a.m.). All the animals housed at L-PCB were maintained with 2914 Irradiated Teklad Global 14% Protein Rodent Maintenance Diet (Harlan) provided *ad libitum*. Autoclaved tap water was also provided *ad libitum*. This test was performed essentially as previously described (Worms et al*.,* 1987). Test compound was administered p.o. at the indicated dose levels to groups of mice (N = 10 / dose) 70 min before an i.p. injection of PEA (25 mg/kg). This dose of PEA produced no effects in control mice. From 20 to 30 min after PEA treatment, mice were observed for the presence of stereotyped behavior. Studies were performed in accordance with the institutional guidelines for the care and use of laboratory animals (European Communities Council Directive 86/609/EEC) established by the Ethical Committee for Animal Experimentation (ECAE) at the PRAAL-PCB. Prior approval was obtained before initiation of the study (Protocol No. 9065; ECAE-PRAAL-PCB).

### Mouse MAO-B inhibition

Male CD1 mice (8 weeks of age, 25-30g) procured from Charles River were housed at animal facility of Renasci. The temperature range was 19-22 ºC with a 35-70 % relative humidity range. The light / dark cycle was maintained at 12:12 hr with switch on at 7:00 a.m.). All the animals housed at Renasci were maintained with normal diet (Harlan) provided *ad libitum*. Autoclaved tap water will be also provided *ad libitum*. Brains were obtained from animals treated, either acutely or chronically, with vehicle, or ORY-2001 by oral gavage. Studies were performed in accordance with the institutional guidelines for the care and use of laboratory animals (European Communities Council Directive 86/609/EEC) established by the Ethical Committee for Animal Experimentation (ECAE) at LEITAT-PCB. Prior approval was obtained before initiation of the study (Protocol No. 9719-LEITAT-PCB).

### Rat tyramine pressure response model

This test was performed essentially as previously described [2]. Male Sprague Dawley (SD) rats for the study were sourced from in-house breeding facility, Advinus Therapeutics Ltd., Pune. Rats were acclimatized to the study area conditions for 1 week before dosing. Animals were housed (one per cage) in polypropylene cages and maintained in controlled environmental conditions with 12 hr light / dark cycles. The temperature and humidity of the room were maintained between 22 ± 3 ºC and 55 ± 5 %, respectively, and approximately 20 fresh air change cycles per hr. LabDiet 5K52 (Manufactured by LabDiet, St. Louis, MO 63144, USA) was provided *ad libitum*. Clean water obtained from Reverse Osmosis system was provided *ad libitum* throughout the study. Rats were implanted with polyethylene catheter in the common carotid artery under urethane anesthesia. Test compound was administered p.o. to rats (N = 6-9 / dose) 60 min after the onset of the anesthesia. Animals were pre-cannulated and 15 mg/kg tyramine was administered orally 1 hr after administration a single oral dose of 3, 10 or 30 mg/kg ORY-2001; or 2 hr after administration of a single dose of 6 mg/kg TCP in the acute or after 5 days of treatment in the subchronic setting. Arterial blood pressure was measured using pressure transducer connected to a polygraph. The tyramine response was expressed as the maximal increase (%) in mean blood pressure (MBP, calculates vs. the pre.tyramine value) within 1 hr after the administration of tyramine. This study was performed as per approved protocol by the Institutional Animal Ethics Committee (IAEC) and in accordance with the requirement of Committee for the Purpose of Control and Supervision of Experiments on Animals (CPCSEA), India. Prior approval was obtained before initiation of the study (Protocol No. T00100 and 152/20012015/PS; IAEC-CPCSEA).

### Mouse L-5-HTP model

Male C57BL/6 mice (8 weeks of age, 25-30 g) procured from Harlan Interfauna Iberica were housed in the same condition that in MAO-B PEA section. This test was performed essentially as previously described [3]. Test compound was administered p.o. at several dose levels to groups of mice (N = 10/dose) 60 min before an i.p. injection of L-5-HTP (100 mg/kg). This dose of 5-L-HTP produced no effects in control animals. Animal were observed for the presence of L-5-HTP induced symptoms from 30 to 40 min after L-5-HTP treatment. Studies were performed in accordance with the institutional guidelines for the care and use of laboratory animals (European Communities Council Directive 86/609/EEC) established by the Ethical Committee for Animal Experimentation at the PRAAL-PCB. Prior approval was obtained before initiation of the study (Protocol No. 9065; ECAE-PRAAL-PCB).

### Mouse MAO-A inhibition

Male CD1 mice (8 weeks of age, 25-30 g) procured from Charles River were housed in the same conditions as in *Ex vivo* assay of MAO-A inhibition section. Animals were treated, either acutely or chronically, with vehicle, or ORY-2001 by oral gavage and animals were sacrificed and brain tissue was obtained for analysis MAO-A inhibition. Studies were performed in accordance with the institutional guidelines for the care and use of laboratory animals (European Communities Council Directive 86/609/EEC) established by the Ethical Committee for Animal Experimentation (ECAE) at LEITAT-PCB. Prior approval was obtained before initiation of the study (Protocol No 9719-LEITAT_PCB).

### SAMR1 and SAMP8 model

Both male and female mice from the senescence accelerated strain SAMP8 and its control SAMR1 of different ages (see Table S3 for details) were used in these studies. Mice were breed in the animal facilities of the Pharmacy Faculty (University of Barcelona), maintained in a at 22 ± 2 ºC controlled temperature in a 12 hr light / dark cycle (lights on at 8:00 am; 300 lux/0 lux) with *ad libitum* access to food (2018 TEKLAD, Harlan) and water. Male mice were housed individually (males) or in groups of 4-5 animals (females) in plastic colony boxes with a sawdust bedding material. Animals were treated at the indicated doses and for the indicated periods with vehicle, ORY-2001, ORY-LSD1 or RSG administered in drinking water (1.8% 2-Hydroxypropyl-b-cyclodextrin, Sigma-Aldrich, Spain). SAMR1 mice treated with vehicle were included as a control. All drugs were administered via drinking water and diluted in vehicle. Drug concentration was calculated weekly in function of body weight and corrected in function of drinking water consumption. Cognitive function was evaluated by NORT, social behavior was evaluated by RI and anxiety and motor function were evaluated by EPM and OF. When necessary, animals were deeply anesthetized (80 mg/kg sodium pentobarbital) and brain samples (hippocampus, (prefrontal) cortex) were micro-dissected and processed according to the downstream analysis. An overview of the SAMP8 studies is provided in Table S3.

Studies were performed in accordance with the institutional guidelines for the care and use of laboratory animals (European Communities Council Directive 86/609/EEC) established by the Ethical Committee for Animal Experimentation at the University of Barcelona. Prior approval was obtained before initiation of the study (Protocol numbers 6946, 10291 and 9861, CEEA-UB). Welfare supervision was conducted by the Veterinarian and the animal care staff.

### Rat isolation rearing model

Sprague Dawley Rats: Right after the weaning (post natal day 21-23), 72 Sprague-Dawley male rats (Charles River, bred in the UAB Animal facility) were divided in two groups: Control (N = 18), maintained 3-4 animals per cage; Isolated (N = 54), 1 animal per cage (800 cm^3^ 1291H Eurostandard type III with ECOPURE CHIPS 6 bedding material, Harlan). Animals were maintained at 21 ± 2 ºC controlled temperature in a 12 hr light / dark cycle (lights on at 8:00 am) with *ad libitum* access to food (2014 TEKLAD, Harlan) and water. Treatment started on postnatal day 61, isolated animals were treated with vehicle, ORY-2001 0.16 mg/kg/day or ORY-2001 0.48 mg/kg/day (N = 18/group) for 5 weeks. Control animals were treated with vehicle. The administration route was drinking water and the vehicle 1.8% 2-Hydroxypropyl-b-cyclodextrin. Drug concentration was adjusted weekly by body weight and water consumption. During the last week of treatment, all animals were tested in the EPM and, on a different day, in the RI test (N = 12 / group). After depth anesthesia with Isoflurane, animals were perfused with saline solution followed by paraformaldehyde in order to obtain brain samples for histological analysis. The experimental protocol was approved by the Ethics Committee at the Universitat Autònoma de Barcelona and the Generalitat de Catalunya, and carried out in accordance to the European Council Directive (2010/63/UE) and Spanish legislation (RD 53/2013). Prior approval was obtained before initiation of the study (Protocol No. 3016 and 3016M2, Comissió d'Ètica en l'Experimentació Animal i Humana _ (CEEAH-UAB). Welfare supervision was made by the Veterinarian staff at weekly basis, by the animal care staff daily and by the researchers 5-7 days/week.

## Method details

### Biochemistry and biophysics

### Inhibition assays

KDM1A IC_50_ values were determined for compounds with >50 % inhibition at 10 µM, as previously described (Yang et al*.*, 2007) with minor modifications. Serial 3-fold dilutions of ORY-2001 ranged between 30 µM and 1 nM were pre-incubated for 15 min with human recombinant KDM1A enzyme (BPS Bioscience, Ref. 50100) on ice in the assay buffer (50 mM sodium phosphate pH 7.4). Each concentration of inhibitor was tested in duplicate. The enzymatic reaction was initiated by the addition of dimethylH3K4 peptide substrate (Anaspec, Ref. 63677), at the appK_M_ of KDM1A. After 30 min of incubation at 37 ºC Amplex Red reagent and the horseradish peroxidase (HRP) solution were added to detect H_2_O_2_ formed in the enzymatic reaction, following the recommendations provided by the supplier (Invitrogen). The mix was incubated for 5 min at room temperature in the dark and the conversion of the Amplex Red reagent to the highly fluorescent resorufin was analyzed with using an Infinite F200 Tecan fluorescence microplate reader (λex = 540 nm, λem = 590 nm). The maximum demethylase activity of KDM1A was obtained in the absence of inhibitor and corrected for background fluorescence in the absence of KDM1A.

Mouse recombinant KDM1B protein was kindly provided by Dr. Andrea Mattevi from the University of Pavia. The biochemical activity assay was carried out at room temperature in a similar manner as for KDM1A but using 50 mM HEPES pH 8.5 as the reaction buffer, but with 10 min of incubation of KDM1B in the presence of dimethylated H3K4 peptide at the K_M_app of KDM1B.

Human recombinant monoamine oxidase proteins MAO-A and MAO-B (Sigma Aldrich, M7316 and M7441) were used to monitor the MAO enzymatic activities in a fluorescent assay using kynuramine as a substrate. Inhibitors were pre-incubated with MAO protein for 15 min on ice in the reaction buffer (100 mM Hepes pH 7.5). Enzymatic reaction was initiated by the addition of specific K_M_ Kynuramine and incubated 1 hr at 37 ºC. The oxidative deamination of the substrate was stopped by adding NaOH 2N (v/v). The conversion of kynuramine to 4-hydroxyquinoline by MAOs was monitored by fluorescence (λex = 320 nm, λem = 360 nm) using a microplate reader. Clorgyline and SLG (Sigma Aldrich, M3778 and M003) were used as controls for specific inhibition of MAO-A and MAO-B respectively; the MAO-A IC_50_ values for inhibition of Clorgyline was 12 nM and the MAO-B IC_50_ for SLG was 9.5 nM. The IC_50_ values for inhibition of KDM1A, MAO-A and MAO-B by TCP were respectively 36 µM, 3 µM and 102 nM in our assays.

Human recombinant IL-4I1 was purchased from RD Systems (Ref. 5684-AO-020). The biochemical activity was measured by its ability to oxidize phenylalanine (specific substrate for IL-4I1; Sigma, P2126) and liberate H_2_O_2_ was measured in a coupled horseradish peroxidase assay using Amplex Red reagent as a substrate, as described in the KDM1A assay. The IL4I1 assay buffer was 50 mM Sodium Phosphate pH 7.0. Inhibition of IL-4I1 was monitored during 30 min in the presence of K_M_ phenylalanine peptide at the K_M_app of IL-4I1.

Mouse recombinant SMOX [4] was provided by Paolo Mariottini (Department of Biology, University of Roma). The biochemical activity was measured by its ability to oxidize spermine (specific substrate for SMOX) and produce H_2_O_2_ using a horseradish peroxidase coupled assay. The assay buffer for the mSMOX reaction was 100 mM Sodium Phosphate pH 8.0. The inhibition was monitored during 10 min in the presence of K_M_ spermine peptide.

**KDM1A splice form kinetics analysis**

Time course experiments were performed to assess the rate of inhibition of demethylation of a H3K4me2 peptide mediated by rKDM1A, monitoring the release of H_2_O_2_ produced during the reaction using a coupled horseradish peroxidase assay using Amplex Red reagent as a substrate. Conditions were the same as for IC_50_ determinations, but all reagents were added simultaneously. K_i_ and k_inact_ were calculated using equations previously described [22](Yang et al*.*, 2007). Kinetic measurements of KDM1A inhibition were obtained for selected compounds with rKDM1A IC_50_ values <200 nM.

ORY-2001 and OG-634 were tested in three different KDM1A splice variants, complexed with RCOR1 (provided by Dr. Claudia Binda, Department of Biology and Biotechnology of the University of PAVIA).

### KDM1A binding analysis

Analysis of binding of inhibitors to KDM1A was as previously described [22](Yang et al*.*, 2007) with minor modifications. The binding to FAD (present in the enzyme or free) was analyzed by measuring the decrease in absorbance at 450nm in a Tecan Infinite F200 microplate reader. Briefly, KDM1A enzyme was mixed with the inhibitor (without substrate) and the absorbance at 450nm was compared with the absorbance of the KDM1A enzyme without inhibitor. The reaction is monitored during 30 min and values used to calculate K_obs._

**MALDI-TOF mass spectrometry**

The sample used to analyze the covalent FAD-ORY-2001 adducts consisted in the reaction mixture of KDM1A (5 mg/ml) and ORY-2001 at 420 µM in 4 M Guanidinium Chloride, 10 mM HEPES pH=7.4, 83 mM NaCl, 2 mM DTT, 0.4 mM PMSF and 2 mM glycerol. The sample was initially purified by reversed phase chromatography in a ZipTipC18 pipette tip (Millipore, USA) wet using column and equilibrated with 0.1% TFA. The sample was bound to the column by repeated aspiring and dispensing to achieve maximum binding. The ZipTip pipette tip was washed three times with 0.1% TFA and eluted in 4 µl. of 50 % ACN/ 0.1% TFA. 1 µl of eluted sample was loaded onto an AnchorChip MALDI target (Bruker, Germany); 2 µl. of matrix solution (0.3 mg/ml HCCA -alpha-cyano-4-hydroxycinamic acid in EtOH/acetone 2:1) was added and the mixture was allowed to dry at room temperature. The sample preparations were analyzed in an AutoFlex Speed MALDI TOF/TOF mass spectrometer (Bruker, Germany), equipped with a Nd:YAG laser. MS spectra were acquired in Reflector mode, with either positive or negative polarity. MSMS post source-decay fragmentation spectra of selected precursor ions were obtained using the LIFT module (Bruker, Germany).

Cell Biology

Cell differentiation in THP-1

THP-1 cells are acute leukemia cells with blast phenotype that can be induced to differentiate into a monocyte/macrophage phenotype by cell penetrant KDM1A inhibitors. Differentiation can be followed by measurement of induction of differentiation surface markers like CD11B. Cells were treated for 96 hr with KDM1A inhibitors. Fluorescent assisted cell sorting (FACS) analysis of CD11B expression was performed on 1.5 x 10^5^ THP-1 cells (obtained from German Collection of microorganism and Cell Cultures; ACC-16) using a phycoerythrin-conjugated human CD11B-specific mouse monoclonal antibody (eBiosciences, #12-0118) at dilution 1:5 on a cytomics FC500 (Beckman Coulter) with FlowJo software. The mouse IgG1 K isotype (eBiosciences, #12-4714) was used as a control antibody. Concentration-response curves were determined out by assaying inhibitors at concentrations ranging between 0.32 and 200 nM.

### Pharmacokinetics

Plasma was harvested from the blood samples harvested in K_2_EDTA containing tubes by centrifugation at 4000 rpm for 10 min at 4 ± 2 ºC and stored below -70 ºC until bioanalysis. Brain tissue was homogenized with phosphate buffer saline (pH 7.4). Total homogenate volume was thrice the tissue weight. All samples were processed for analysis by precipitation using Imipramine as internal standard and analyzed with partially validated LC-MS/MS method. Pharmacokinetic parameters were calculated using the non-compartmental analysis tool of WinNonlin® Enterprise software (Pharsight Corp., USA; version 5.2).

### Pharmacodynamics

**Hematology**

Blood samples obtained from leg saphenous vein or cardiac puncture were analyzed on a Diatron hematology analyzer at SEA (Plataforma Recerca Aplicada Animal de Laboratori, Parc Científic de Barcelona).

***Ex vivo* analysis of KDM1A target engagement**

Native protein extracts of cells or tissues were obtained in presence of 25 nM OG-881 chemoprobe according the protocol described in Mascaró et al. [25,26]. Total and free KDM1A were measured by Luminescent ELISA, and the percentage of target engagement of ORY-2001 to KDM1A was calculated relative to the vehicle treatment.

Luminescent ELISA: for quantification of total amount of KDM1A protein (TOTAL KDM1A), Luminunc Plates Maxisorp (NUNC; #436110) were coated with a monoclonal anti-KDM1A antibody (Abcam; #ab53269) at 2 µg/mL in PBS. For quantification of the fraction not bound to inhibitor (FREE KDM1A), plates were coated with streptavidin (Promega Biotech Ibérica; #Z7041) at 10 µg/mL in PBS. Coating was performed at 4°C O/N. Wells were washed 3 times in PBS, 0.1% Tween-20 and blocked with PBS-BSA 1% (Sigma; #A3059) for 2 hr. A KDM1A calibration curve of full length rKDM1A (Active Motif; #31334) diluted in PBS was included in each plate. Plates were then incubated 1 hr at RT and washed 5 times. Afterwards, monoclonal anti-KDM1A antibody (Cell Signaling; #2184) was diluted at 0.125 µg/mL in PBS and plates were incubated 1 hr at RT. After 6 washes, a peroxidase-conjugated secondary antibody (Jackson Inmunoresearch; #711-035-152) diluted 1:5000 was added to plates, incubated for 1 hr at RT and plates were washed again. 100 µL/well of chemiluminescent substrate (Invitrogen; # 37074) was added. Plates were centrifuged during 30 sec to 1,500 x g, shaken for 1 min at 100rpm and incubated inside the micropate reader (Infinite 200, Tecan) for 3 min at 25^0^ C. The relative luminescence units (RLU) readouts were acquired using a 1000 msec integration time and 150 msec settle time.

**MPTP toxicity test**

48 hr after administration of MPTP (or blank), animals were tested for locomotor activity in an Open Field Test by placing them into a 45 x 45 cm open chamber and recording the movement using Smart Junior and reporting locomotor activity at 5 min intervals over a 20 min period. Data were presented as distance travelled (cm) for horizontal locomotor activity and vertical rearing. TH immunohistochemistry was performed on the SNc (substancia nigra compacta) of control and treated animals from the MPTP toxicity assay.

**PEA-induced symptoms test**

This test was performed essentially as previously described (Worms et al*.*, 1987). ORY-2001 was administered p.o. 70 min before an i.p. injection of PEA. From 20 to 30 min after PEA treatment, mice were observed for the presence of stereotyped behavior, using the following rating scale: 0, normal behavior; 1, sniffing without mouth movement; 2, mouth movement. Thus the maximal obtainable score was 20 per group of 10 mice. The effect of the compound was expressed as ED_50_ defined as the dose that provided 50 % of maximal obtainable score.

***Ex vivo* analysis of MAO-B inhibition**

The ability of ORY-2001 to inhibit monoamine oxidase B in the CD1 mouse brain after acute and chronic treatment was evaluated. Ex-vivo MAO-B activity was measured essentially as previously described (Kilpatrick et al*.*, 2001; Youdim and Tipton, 2002). Brains obtained from animals treated, either acutely or chronically, with vehicle, or ORY-2001 were weighed, thawed and homogenised in 20 volumes of homogenisation buffer, after which MAO-B activity was measured. MAO-B activity was determined over 20 min using 10 μM β‑phenylethylamine hydrochloride [ethyl-1-^14^C] following a 20 min preincubation of homogenate with 100 nM clorgyline. Enzyme activity was corrected by subtracting an assay buffer blank and initially expressed in units of nmoles substrate/min. The protein concentration of the homogenates was measured and used to express final enzyme activity in units of nmoles/min/mg protein. All results were expressed as a mean of duplicate determinations. Brains from animals treated acutely were assayed on a different day to those treated for 5 days (chronically).

**Tyramine pressure response test**

This test was performed essentially as previously described (Kato et al*.*, 1998). Test compound was administered p.o. to rats (N = 6-9 / dose) 60 min after the onset of the anesthesia. Animals were pre-cannulated and 15 mg/kg tyramine was administered orally 1 hr after administration a single oral dose of 3, 10 or 30 mg/kg ORY-2001; or 2 hr after administration of a single dose of 6 mg/kg TCP in the acute or after 5 days of treatment in the subchronic setting. Arterial blood pressure was measured using pressure transducer connected to a polygraph. The tyramine response was expressed as the maximal increase (%) in mean blood pressure (MBP, calculates vs. the pre.tyramine value) within 1 hr after the administration of tyramine.

**L-5-HTP-induced symptoms test**

This test was performed essentially as previously described [90](Koe et al*.*, 1983). Test compound was administered p.o. at several dose levels 60 min before an i.p. injection of L-5-HTP. Animal were observed for the presence of tremor, hindlimb abduction and head twitch from 30 to 40 min after L-5-HTP treatment. One score assigned to each symptom observed per animal, giving a maximal obtainable score of 30 per group of 10 mice. The effect of a test compound was expressed as ED_50_, defined as the dose that provided 50 % of the maximal obtainable score.

***Ex vivo* analysis of MAO-A inhibition**

Ex-vivo MAO-A activity was measured essentially as previously described [5, 6] . Animals were treated, either acutely or chronically, with vehicle, or ORY-2001. Brain samples were weighed and then thawed and homogenised in 20 volumes of homogenisation buffer after which MAO-A activity was measured. MAO-A activity was determined over 20 min using 10 μM serotonin creatinine sulphate [2‑^14^C] following a 20 min preincubation of homogenate with 1 μM SLG. Enzyme activity was corrected by subtracting an assay buffer blank and initially expressed in units of nmoles substrate/min. The protein concentration of the homogenates was measured and used to express final enzyme activity in units of nmoles/min/mg protein. All results are expressed as a mean of duplicate determinations. Brains from animals treated acutely were assayed on a different day to those treated for 5 days (chronically).

### Efficacy studies

**Novel Object Recognition Test (NORT)**:

The protocol was performed as previously described [7]. Briefly, animals were placed in a 90°- two-arm maze. Mice were habituated to the apparatus for 10 min during 3 consecutive days. On day 4, two identical novel objects (A+A or B+B) were placed at the end of each arm and animals performed a 10-min acquisition trial. A 10-min retention trial was carried out 2 hr after the acquisition trial with object A and B. Time exploring the novel (NO) and the familiar object (FO) was measured and a discrimination Index (DI) was defined as (NO-FO)/(NO+FO). In some experiments, a more restrictive threshold was applied and animals exploring less than 5 sec (NO+FO) were excluded from the analysis (Table S3).

**Open Field (OF):**

A 50 × 50-cm white plastic arena with 25-cm-high walls was used to analyze spontaneous exploratory behavior. The floor of the apparatus was divided into 25 equal squares. The movements of each animal were video recorded during 5 min. Locomotor activity was analyzed by video-tracking over the captured images using SMART® (v 2.5.21 (rat) or v3.0 (mice), PanLab, SLU, Spain).

**Elevated Plus Maze (EPM):**

The EPM consisted of four arms at right angles to each other connected to a central square and maintained elevated 50 cm above the floor. Two of the opposite arms had high walls (enclosed arms, 30 x 5 x 15 cm for mice and 46.5 x 12 x 42 cm for rat), whereas the other two were open arms (30 x 5 x 0 cm for mice and 46.5 x 12 x 0.3 cm for rat). The animal was placed facing a closed arm, and its movements were video-recorded for 5 min and analyzed by video-tracking using SMART® (v3.0, PanLab, SLU, Spain).

**Resident Intruder (RI):**

The protocol was modified from previously described [8]. Briefly, the test subject (resident) was maintained in its home cage without bedding changes for one week. On the test day, a significantly younger and smaller subject (intruder; 50 days old Sprague-Dawley rat or 90 days old C57BL6 mice) was introduced in the resident home cage. In mice the session was video-recorded for 20 min and social interaction (social interaction and rearings) and aggressive behavior (lateral threats, clinch attacks, keep-down behavior) were analyzed. “Normal” aggressive behavior usually starts after a period of social exploration that includes rearing (the animal stand on its rear limbs exploring the intruder from the distance) direct sniffing, visual evaluation and some physical contact. “Attacks” are from different nature, usually starting with a lateral threat where the resident blocks the intruder by situating the side of its body in front of the head of the intruder. This might be followed by a clinch attack where the resident actively uses its limbs to immobilize and bite the intruder. As a result a keep down behavior may follow a clinch attack, when the resident maintains the intruder immobilized with its back facing the floor. The total number of attacks is the sum of lateral threats, clinch attacks and keep down behavior. In rats the session was video recorded for 15 min and social interaction (active and passive social interaction, number of evitations and time without social interaction) were analyzed. The social interaction was divided in active, when the resident performed the exploration behavior of the intruder, and passive when resident was explored by the intruder. Social interaction included sniffing, visual evaluation and some physical contact. When the resident actively avoided the interaction with the intruder it was considered as an evitation. In addition, the time spent without social interaction was also measured.

**Three Chamber Test (TCT):**

The test was performed in a Plexiglas transparent box with three identical consecutive chambers (15 x 15 x 20 cm). Adjacent chambers were communicated and animals were free to move from one to another. The test subject (SAMR1 or SAMP8 female mice) was allowed to explore the empty apparatus for 5 min. This habituation time was monitored to avoid animals showing preference for one of the chambers. Then two small metallic cages were placed in both lateral chambers, one containing a novel female mouse (Mice Chamber) and the other remaining empty (Object Chamber). The time spent in each chamber and the time of direct exploration of the novel mice was measured.

Blinding: SAMR1 and SAMP8 mice: memory (NORT) and aggression tests (RI) of each mouse, were recorded by using a video camera. The principal researcher performed the experiments and recorded the original videos. A second (supporting) researcher made an index of the files and recoded the complete video dataset. The principal researcher analyzed all the video files and built the codified raw data. The supporting researcher decoded the raw data and after this, statistical analyses were performed. Blinding was applied in all SAMP8 experiments except Exp1 and Exp2 of Table S3. Sprague-Dawley rat behavior tests: experimenters evaluating animal behavior were blind to the treatment. Blinding was performed as follows: the day before the social defeat test, an experimenter unaware of the treatments of the animals recoded the number of the rat and planned the order of the testing for the next day (counterbalancing the order of the groups). Another different experimenter analyzed the tapes and after the behavioral scoring the code was opened.

### Gene expression analysis

**Oligo design, microarray fabrication and quality control**

DNA microarray oligo probes were designed using *Tethys*, Oryzon's proprietary software which is based on the *in silico* thermodynamic simulation of hybridization. Agilent probes were added for genes which yielded no probes complying *Tethys* criteria. Control probes were used to assess detection limits and range, to verify spatial homogeneity, and to determine experimental within-array variation. Microarray slides were synthesized by Agilent.

**RNA extraction, microarray hybridization and analysis**

Total RNA was extracted using the RNeasy extraction kit (Qiagen, 74106). Evaluation of RNA quality was performed with the Agilent 2100 bioanalyzer and NanoDrop™ ND-1000 (Thermo Scientific). Total RNA (0.5 μg) amplification and labeling with Cy3 or Cy5 was carried out using a modified Eberwein mRNA amplification procedure employing the MessageAmp™ aRNA amplification kit from Ambion (Applied Biosystems). The Cy3- and Cy5-labelled cRNA mixes were hybridized to microarrays according to the manufacturer’s instruction. Raw data were obtained using Agilent's DNA Microarray Scanner G2505B and Feature Extraction software (v10.1) and processed using the proprietary *Polyphemus* software. Data were normalized by modified nonlinear *Q-splines* normalization method and Log_2_(sample/control) values calculated without background correction (which permits robust selection of differentially expressed genes yet may lead to sub-estimation of the magnitude of change for genes expressed near the detection limit). Differential expression was assessed with Polyphemus using robust statistics on the average technical replicates (3 replicates gene oligo datapoint) after removing eventual outlier points (caused by dust or array imperfections). *Polyphemous* automatically defines the criteria for outlier elimination by assessing the intra-array technical variability using the signal distribution of controls probes (a large number of replicates present on the array). The p-values were calculated after outlier elimination based on the absolute value of the regularized t-statistics, which uses a Bayesian framework to derive the algorithm, using internal replicated controls to assess the minimum technical variability of the process.

**qRT-PCR**

Total RNA was extracted using the RNeasy extraction kit (Qiagen). First strand synthesis was performed with the High Capacity RNA to cDNA Master Mix (Invitrogen, 4390779). qRT-PCR reactions were performed in triplicate (N = 3) using 10-20 ng of 1^st^ strand DNA and off-the-shelf Taqman assays, in a Roche Lightcycler II 480. Taqman assays used were: Gapdh: Mm_99999915_g1; Gusb: Mm_03003537_s1; S100a9: Mm_00656925_m1; Baiap3:_Mm 01301145_m1; Npw: Mm_01299908_g1; Prph: Mm_00449704_m1; Fos: Mm_00487425_m1; Npas4; Mm_01227866_g1; Calb2: Mm_00801461_m1; Gng4: Mm_00772342_m1; Ccl19: Mm_00839967_g1; Baiap3: Mm_01301145_m1.

### Protein expression and interaction analysis

**Western blot and Silver Nitrate staining**

Samples were separated on a 10% Bis-Tris NuPAGE Novex Precast gel (Invitrogen; Cat# NP0301BOX) following manufacturer’s instructions. For immunodetection, gels were transferred onto a nitrocellulose membrane by dry transfer (Cat# IB23001, Thermo Fisher Scientific). N-specific binding sites were blocked by incubating membranes in blocking buffer (5% non-fat dry milk in PBS-Tween 0.1%) at RT for 1 h. Nitrocellulose membranes were then incubated overnight at 4°C with an anti-KDM1A antibody (Cat# 2184, Cell Signaling) diluted 1:50,000 or an anti-RCOR1 antibody (Cat# ab56165, Abcam) diluted 1:800 in 5% milk in PBS-Tween 0.1%. After 3 washes in PBS-Tween 0.5%, the membrane was incubated for 1 hr at RT with a peroxidase-conjugated donkey anti-rabbit antibody (Cat# 711-035-152, Jackson Inmunoresearch). Signal was detected by enhanced chemiluminescence (ECL, Amersham, GE Healthcare; Cat# RPN2232) using the G:BOX CHEMI XRQ System (Cat# SE-GBOX-CHEMI-XRQ, Syngene). For Silver Nitrate staining, acrylamide gels were incubated with fixing solution (40% Ethanol, 10% Acetic Acid in milliQ H_2_O) for 30 min at RT. Gels were then incubated with sensitizing solution (8 mM Sodium Thiosulfate, 500 mM Sodium Acetate, 30% Ethanol in mqH_2_O) for 30 min at RT. After 3 washes in mqH_2_O, gels were subsequently stained with 15 mM Silver Nitrate in mqH_2_O for 20 min at RT. Following 2 washes with mqH_2_O, gels were incubated with 235 mM Sodium Carbonate in milliQ H_2_O for 2-5 min at RT. Finally, the reaction was stopped by incubating the gel with a 40 mM EDTA solution for 10 min at RT.

**Chemoproteomics**

3 x 10^6^ SH-SY5Y cells were pelleted and frozen at -80°C until further processing. For the pulldown, each cell pellet was lysed in 200 μL of Cell Lysis Buffer (Cat# 9803, Cell Signalling) supplemented with Complete mini-Protease Inhibitor Cocktail (Cat# 11836153001, Sigma-Aldrich) and with 100 nM biotinylated KDM1A chemoprobe (OG-881). Samples were maintained in ice for 5 min prior to sonication and high speed centrifugation to separate cellular debris. Cleared lysates were incubated 1 hr on ice, to allow binding of the chemoprobe. For the pulldown, 150 μL of Dynabeads™ M-280 Streptavidin beads (Cat# 11205D, Thermo Fisher Scientific) were added to 450 μg of protein in a total volume of 300 μL of Cell Lysis Buffer and incubated for 30 min at room temperature on a spinning wheel. As negative controls, cells were incubated with beads in the absence of the OG-881 chemoprobe. After magnetic isolation, the beads were washed 5 times with ice-cold PBS to remove non-specific binding. Elution was performed using 25 μL of 1% SDS, incubating the beads for 15 min at 95°C. The eluted protein was reduced with DTT (dithiothreitol) at a final concentration of 10 mM and incubated for 30 min at 60°C. Subsequently, the samples were treated with 55 mM Iodoacetamide for 30 min at RT, protected from light. Eluted protein was precipitated with 10% Trichloroacetic Acid, left on ice for 15 min and centrifuged at 16,100 rcf for 15 min at 4ºC. The resulting pellet was then resuspended in 8 M urea in 50 mM Tris pH 8.0 and digested with porcine trypsin (1 μg) overnight at 4ºC. The reaction was stopped with Formic Acid at a final concentration of 0.1%. Finally, peptides were analyzed on reverse-phase nanoLC chromatography (nano Ultra 2D Eksigent) coupled to a LTQ-Velos-Orbitrap mass spectrometer (Thermo Scientific). Mass spectrometry analysis was performed at the Proteomics Unit CCIT-UB at the University of Barcelona.

**Chemoprobe ELISA**

5-10 x 10^6^ SH-SY5Y cells were pelleted and frozen at -80°C until further processing. The day before the assay, plates were coated with streptavidin (Cat# Z7041, Promega Biotech Ibérica) at 10 µg/mL in PBS at 4°C O/N. On the day of the assay, cell pellets were thawed and resuspended in 400 μl Cell Lysis Buffer (Cat# 9803, Cell Signalling) supplemented with Complete mini-Protease Inhibitor Cocktail (Cat# 11836153001, Sigma-Aldrich) and 25 nM OG-881 chemoprobe. Samples were maintained on ice for 10 min prior to high speed centrifugation to remove cellular debris and the supernatant kept on ice. Wells were washed 3 times in PBS 0.1% Tween-20 and blocked with PBS-2% milk for 2 hr. Samples were added to the plate (44, 25 and 2.5 μg of total protein per well) and the plate was incubated 1 hr at RT to allow capturing of the chemoprobe tagged KDM1A complex. Washing and detection was performed as in the KDM1A interaction ELISAs below, using a mouse anti-HDAC1(Cat# 5356, Cell Signaling) antibody at 0.2 μg/mL and an anti-HDAC2 (Cat# 5113, Cell Signaling) antibody at 0.02 μg/mL. The chemoprobe ELISA is available at

**KDM1A Interaction ELISAs**

5-10 x 10^6^ SH-SY5Y cells were pelleted and frozen at -80°C until further processing. The day before the assay, Luminunc Plates Maxisorp (Cat# 436110, NUNC) were coated with the 100 μl/well of the capture antibody and incubated O.N. at 4°C. Mouse antibodies were used to coat the plates and rabbit antibodies were used as primary detection antibodies. On the day of the assay, cell pellets were thawed and resuspended in 400 μl Cell Lysis Buffer (Cell Signalling) supplemented with Complete mini-Protease Inhibitor Cocktail (Cat# 11836153001, Sigma-Aldrich). Samples were maintained on ice for 10 min prior to high speed centrifugation to remove cellular debris. The plate coated O.N. was then washed 3 times with washing buffer (0.1% Tween-20 in PBS 1X) and blocked in PBS + 2% Milk for 1 hr at RT. Samples were added to the plate (SH-SY5Y: 44, 25 and 2.5 μg of total protein per well; human hippocampus: 80, 30 and 10 μg of total protein per well) and the plate was incubated 1 hr at RT. The plate was washed 5 times with washing buffer, detection antibodies diluted in PBS were added and the plate incubated for 1 hr at RT. Wells were washed 6 times with washing buffer, incubated with secondary peroxidase-conjugated donkey anti-rabbit IgG antibody (Cat# 711-035-152, Jackson Immunoresearch) for 1 hr at RT and washed 6 times again. Following the last wash, 100 μL/well of chemiluminescent substrate (Cat# 37074, Thermo Fisher Scientific) were added to the plates. Plates were centrifuged 30 secs to 1,500 rpm in order to eliminate bubbles and shaken during 1 minute at 100 rpm. The luminescence was measured using an Infinite 200-Tecan Microplate reader. Plates were incubated inside the reader at 25 °C for 3 min and luminescence read at 1000 msec integration time and 150 msec settle time.

The following antibody combinations were used:

Capture antibody: mouse anti-KDM1A (Cat# ab53269, Abcam) with the following rabbit primary detection antibodies: anti-GFI1 (Cat# ab21061), anti-SRF (Cat# ab53147), anti-RCOR1 (Cat# ab56165), anti-OVOL2 (Cat# ab83265), anti-RCOR3 (Cat# ab76921), anti-REST (Cat# ab75785) from Abcam; anti-CtBP1 (Cat# 8684) and anti-GTF2I (Cat# 4562, used on SH-SY5Y cells) from Cell Signaling; anti-SIRT1 (Cat# 07-131) from Merck Millipore; anti-RCOR2 (Cat# 23969-1-AP) from Proteintech; anti-ZMYM3 (Cat# HPA003211), anti-ZNF217 (Cat# HPA051857), anti-GTF2I (Cat# HPA026638, used for the ELISA on human hippocampal tissue), anti-ZMYM2/ZNF198 (Cat# HPA031765), anti-SVIL (Cat# S8695), anti-ZEB1 (Cat# HPA027524), and anti-MYT1 (Cat# HPA006303) from Sigma; or in the vice versa combination: mouse capture antibodies: anti-HDAC1 (Cat# 5356) and anti-HDAC2 (Cat# 5113) from Cell Signaling; SNAI1 (Cat# sc-271977) and ZNF326 (Cat# sc-390606) from Santa Cruz Biotechnology in combination with the rabbit anti-KDM1A C69G12 antibody (Cat# 2184, Cell Signaling). All antibodies were used at a final concentration of 1 μg/mL with the exception of anti-HDAC1 (2 μg/mL), anti-HDAC2 (0.5 μg/mL), anti-SNAI1 (2 μg/mL), anti-ZNF326 (2 μg/mL), anti-CTBP1 (1/100, unknown stock concentration).

**Double labeling immunofluorescence**

Double-labeling immunofluorescence and confocal microscopy was carried out on de-waxed sections, 4 microns thick, stained with a saturated solution of Sudan black B for 15 min to block autofluorescence of lipofuscin granules present in cell bodies; and rinsed in 70 % ethanol and washed in distilled water. The sections were boiled in citrate buffer to enhance antigenicity and blocked for 30 min at room temperature with 10 % fetal bovine serum diluted in PBS. Then, the sections were incubated at 4 ºC overnight with combinations of primary antibodies against S100A9 and anti-phospho-tau (MN1020 Thermo scientific) or anti-β-amyloid (M0872 DAKO). After washing, the sections were incubated with Alexa488 or Alexa546 (1:400, Molecular Probes) fluorescence secondary antibodies against the corresponding host species. Nuclei were stained with DRAQ5TM (1:2,000, Biostatus). After washing, the sections were mounted in Immuno-Fluore mounting medium (ICN Biomedicals, USA), sealed, and dried overnight. Sections were examined with a Leica TCS-SL confocal microscope.

**S100A8/S100A9 ELISA**

96-well white plates (Nunc, 436110) were coated overnight at 4 ºC with anti MRP8/14 antibody (Abcam, ab50138), plates were washed 3 times with wash solution (PBS-Tween 0.1%) and subsequently blocked for 3 hr with PBS + 2% milk blocking solution. Blocking solution was removed and plates were washed 3 times with wash solution. Samples and a dilution series of recombinant MRP8/MRP14 for the standard curve were then added to individual wells and incubated for 1 hr at room temperature. Samples were removed, plates were washed 5 times with wash solution and incubated with primary anti-S100A9 detection antibody (Atlas Antibodies, HPA004193) diluted in PBS + 2% milk for 1 hr at room temperature. Primary antibody solution was removed, plates washed 6 times with wash solution, and incubated for 1 hr at room temperature with HRP-conjugated donkey secondary anti-rabbit antibody (ThermoScientific, 31458) diluted in PBS + 2% milk. Plates were washed 6 times with wash solution and 1:1 SuperSignal ELISA Femto Luminol/Enhancer Solution and SuperSignal ELISA Femto Stable Peroxide Solution - Super Signal ELISA Femto Maximum Sensitivity (Thermo Scientific, 37074) was added to the plate. Luminescence signal was collected as Relative Light Units (RLUs) in a TECAN Infinite F200 plate reader using an integration time of 1000 ms and a settle time of 150 ms.

## Quantification and statistics

### Biochemical assays

The inhibition of the FAD cofactor containing enzymes KDM1A, KDM1B, IL4I1 and SMOX was determined measuring the release of H_2_O_2_ at the respective end points as described and calculating the IC_50_ values using Graphpad Prism5 Software (<https://www.graphpad.com/>), if within dose range.

The inhibition of the FAD cofactor containing enzymes MAO-A and MAO-B was determined measuring the conversion of kynuramine to 4-hydroxyquinoline at the respective end points as described and calculating the IC_50_ values using Graphpad Prism5 Software, if within dose range.

Data for KDM1A, MAO-B and MAO-A for ORY-2001 represent a mean IC_50_ ± SD from minimum 2 independent experiments.

**KDM1A splice form kinetic analysis**

The kinetics of the inhibition of the KDM1A splice forms was analyzed measuring the release of H2O2 in function of time as described and calculating K_i_ and k_inact_ using equations described previously (Yang et al*.*, 2007).

**KDM1A binding analysis**

Absorbance at 450 nM was measured as described and K_obs_ was calculated following the equation:

$product=\frac{vo\left( 1-e^{-kt} \right)}{k}+ offset$

where

$$product= \Delta Abs(450nm)\times1000$$

**Cell differentiation in THP-1:**

Concentration-response curves were determined out by assaying inhibitors at concentrations ranging between 0.32 and 200 nM and IC_50_ values calculated using Graphpad Prism5 Software.

**Pharmacokinetics**

Compound levels from 3 samples per timepoint (for a given dose and route of administration), were used to calculated pharmacokinetic parameters using the non-compartmental analysis tool of WinNonlin® Enterprise software (Pharsight Corp., USA; version 5.2).

**Pharmacodynamics**

**Hematology**

Hematology parameters were calculated as mean ± SEM and represented as a timecourse.

***Ex vivo* KDM1A target engagement:**

The target engagement (TE) or percentage of KDM1A bound by ORY-2001 was calculated using a relative quantification method in which the target engagement in a given sample X was calculated relative to a reference sample REF (pre-treatment or vehicle). All assays were run as triplicate technical replicates (n = 3). After raw data processing (robust elimination of outliers according to Grubbs criteria and subtraction of blank signal on technical replicates), the target engagement was calculated as

$TEx\left( \% \right)=100-(\left( \frac{Rx\left( \% \right)}{Rref\left( \% \right)} \right)\times100)$; where $R\left( \% \right)=\frac{RLUFree}{RLUTotal}\times100$,

where RLUFree and RLUTotal were the mean values from n technical replicates from N animals.

**MPTP neurotoxicity assay**

Locomotor activity was recorded as outlined above. Mean and SEM are represented. Iglewicz and Hoaglin's robust test for multiple outliers was applied prior to statistical analysis with modified Z factor 3. The decision to eliminate these animals as outliers was further supported by the TH readout.

**Analysis of PEA-induced symptoms**

Stereotyped behavior was rated as outlined above. The effect of the compound was expressed as ED_50_ defined as the dose that provided 50 % of maximal obtainable cumulative score.

***Ex vivo* analysis of MAO-B inhibition**

MAO-B activity was determined using β‑phenylethylamine hydrochloride [ethyl-1-^14^C] as outlined above. Raw enzyme activity values (in nmoles substrate/min) were corrected by subtracting an assay buffer blank and were normalized to the total protein concentration (in nmoles/min/mg protein). All results are means of duplicate determinations. Different drug treatments were compared by Oneway-ANOVA with Dunnett analysis.

**Analysis of the tyramine pressure response**

The tyramine response was expressed as the maximal increase (%) in mean blood pressure (MBP, calculates vs. the pre-tyramine value) within 1 hr after the administration of tyramine. Mean and SEM were calculated. Different drug treatments were compared by Oneway-ANOVA with Dunnett analysis.

**Analysis of L-5-HTP-induced symptoms.**

Behavior was rated as outline above. The effect of a test compound was expressed as ED_50_, defined as the dose that provided 50 % of the maximal obtainable cumulative score.

***Ex vivo* analysis of MAO-A inhibition**

MAO-A activity was determined using serotonin creatinine sulphate [2-^14^C] as outlined above. Raw enzyme activity values (in nmoles substrate/min) were corrected by subtracting an assay buffer blank and were normalized to the total protein concentration (in nmoles/min/mg protein). All results are means of duplicate determinations. Different drug treatments were compared by Oneway-ANOVA with Dunnett analysis.

**Efficacy studies**

**Functional testing**

Novel Object Recognition Test (NORT): Time exploring the novel (NO) and the familiar object (FO) was measured and a discrimination Index (DI) was defined as (NO-FO)/(NO+FO). In some experiments, a more restrictive threshold was applied and animals exploring less that 5 sec (NO+FO) were excluded from the analysis (Table S3). Open Field (OF): Locomotor activity was analyzed by video-tracking over the captured images using SMART® (v 2.5.21 (rat) or v3.0 (mice), PanLab, SLU, Spain).

Elevated Plus Maze (EPM): Movements were video-recorded for 5 min and analyzed by video-tracking using SMART® (v3.0, PanLab, SLU, Spain).

Resident Intruder (RI): Mice were video-recorded for 20 min and social interaction and aggressive behavior were analyzed. Rats were video recorded for 15 min and social interaction (active and passive social interaction, number of evitations and time without social interaction) were analyzed.

Three Chamber Test (TCT): Mice were recorded for 5 min during habituation and an additional X min to run the test. The time spent in each chamber and the time of direct exploration of the novel mice was measured.

Means and SEM were calculated. SAMR1 and SAMP8 were compared by Student t-test. Within SAMP8 cohorts drug treatments were compared by one-way-ANOVA with Dunnett and SNK post-hoc analysis. N = number of animals, F = F statistics, t = t-statistics and p = p values are represented *p<0.05, **p<0.01, ***p<0.001.

**GE analysis**

Microarray analysis: Raw data were obtained using Agilent's DNA Microarray Scanner G2505B and Feature Extraction software (v10.1) and processed using the proprietary *Polyphemus* software. Data were normalized by modified nonlinear *Q-splines* normalization method and Log_2_(sample/control) values calculated without background correction (which permits robust selection of differentially expressed genes yet may lead to sub-estimation of the magnitude of change for genes expressed near the detection limit). Differential expression was assessed with Polyphemus using robust statistics on the average technical replicates (3 replicates gene oligo datapoint) after removing eventual outlier points (caused by dust or array imperfections). *Polyphemous* automatically defines the criteria for outlier elimination by assessing the intra-array technical variability using the signal distribution of controls probes (a large number of replicates present on the array). The p-values were calculated after outlier elimination based on the absolute value of the regularized t-statistics, which uses a Bayesian framework to derive the algorithm, using internal replicated controls to assess the minimum technical variability of the process.

**qRT-PCR**

The mean Cp value for each sample datapoint S (Sample) was calculated after outlier elimination (applied if the SD of the three technical PCR replicates was > 0.25). Data were presented as ΔCp values relative to the endogenous control (Figure 2e-i, 4g):

$$\Delta Cp=\left( Cp gene-Cp endogenous \right)S$$

qRT-PCR data for stress response were represented as -ΔΔCp values relative to the endogenous control and the average of the vehicle treated basal SAMP8 samples as the reference sample REF (Figure 4i-l).

$-\Delta\Delta Cp=\left( Cp gene -Cp endogenous \right)S -\left( \bar{Cp gene-Cp endogenous} \right)REF$.

Fold changes (FC) were calculated as $FC= 2^{\left[ \left( -\bar{\Delta\Delta Cp stress} \right) -\left( \bar{-\Delta\Delta Cp basal} \right) \right]}$.

Stress to basal comparisons were done using two-way ANOVA with Bonferroni post-hoc analysis. Analyses of basal or stress levels relative to the vehicle-treated SAMP8 condition were done using one-way ANOVA with Dunnett’s multiple comparison test.

**Chemoproteomics**

The list of nuclear proteins isolated using the KDM1A-specific chemoprobe were visualized using String (http://string-db.org/; Szklarczyk et al., 2015), to generate the final graphical representation of the protein networks.

**Chemoprobe and KDM1A interaction ELISAs**

For each antibody, the signal/background ratio was calculated by dividing the values of every condition by the average of the antibody blank values. The Log_2_ values were then calculated.

**S100A8/A9 ELISA**

The mean and standard deviation of the Blank (no sample) RLUs were calculated after outlier elimination using the Grubbs’ test if the CV of the technical triplicates > 25%. The Lower Limit of Quantification of was calculated as

LLOQ = Mean_Blank_ + 10 x SD_Blank_.

All data points with RLUs below the LLOQ were excluded from subsequent analyses. The Mean_Blank_ value was subtracted from data points above the LLOQ and outliers were eliminated for all data points using the Grubbs’ test (applied if the CV of technical triplicates was > 15%). A 4^th^ order polynomial regression model was applied to the RLU data of the standard curve and this was used to interpolate the S100A8/A9 heterodimer concentration of each sample. Normality was tested by D'Agostino & Pearson omnibus normality test and since the data did not pass the normality test statistical significance was calculated by Mann-Whitney test. ***p < 0.001.

## Data deposition and software

## The data corresponding to the figures in the manuscript have been deposited at Mendeley with doi:10.17632/sc5tfwt4nr.1. Microarray data have been submitted to NCBI-GEO under accession number GSE100413. Polyphemous is proprietary software of Oryzon Genomics S.A. and implemented inhouse as a hardware-embedded microarray pipeline management tool. It is not available as an exportable .exe code

## Additional resources

## Additional statistical data on behavior tests and biomarkers are available upon request. References

1. Tsuchiya S, Yamabe M, Yamaguchi Y, Kobayashi Y, Konno T, Tada K. Establishment and characterization of a human acute monocytic leukemia cell line (THP-1). International Journal of Cancer. 1980;26(2): 171–176.

2. Kato M, Katayama T, Iwata H, Yamamura M, Matsuoka Y, and Narita H. In vivo characterization of T-794, a novel reversible inhibitor of monoamine oxidase-A, as an antidepressant with a wide safety margin. J Pharmacol Exp Ther. 1998;284: 983-990.

3. Koe BK, Weissman A, Welch WM, and Browne RG. Sertraline, 1S,4S-N-methyl-4-(3,4-dichlorophenyl)-1,2,3,4-tetrahydro-1-naphthylamine, a new uptake inhibitor with selectivity for serotonin. J Pharmacol Exp Ther. 1983;226*:* 686-700.

4. Cervelli M, Polticelli F, Federico R and Mariottini P.Heterologous expression and characterization of mouse spermine oxidase. J. Biol. Chem. 2003;278: 5271-5276.

5. Kilpatrick IC, Traut M, and Heal DJ. Monoamine oxidase inhibition is unlikely to be relevant to the risks associated with phentermine and fenfluramine: a comparison with their abilities to evoke monoamine release. Int J Obes Relat Metab Disord. 2001;25: 1454-1458.

6. Youdim MB, and Tipton KF. Rat striatal monoamine oxidase-B inhibition by l-deprenyl and rasagiline: its relationship to 2-phenylethylamine-induced stereotypy and Parkinson's disease. Parkinsonism Relat Disord. 2002;8*:* 247-253.

7. Griñan-Ferré C, Puigoriol-Illamola D, Palomera-Avalos V, Perez-Caceres D, Companys-Alemany J, Camins A, et al. Environmental Enrichment Modified Epigenetic Mechanisms in SAMP8 Mouse Hippocampus by Reducing Oxidative Stress and Inflammaging and Achieving Neuroprotection. Front Aging Neurosci. 2016;8: 241.

8. Saudou F, Amara DA, Dierich A, LeMeur M, Ramboz S, Segu L, et al. Enhanced aggressive behavior in mice lacking 5-HT1B receptor. Science. 1994;265: 1875-1878.
