# Supplementary material for: Modulation of KDM1A with vafidemstat rescues memory deficit and behavioral alterations
Source: PLoS One. 2020 May 29;15(5):e0233468. doi: 10.1371/journal.pone.0233468 (PMC7259601; doi:10.1371/journal.pone.0233468)
Supplement: Attachment — Submitted filename: Response to reviewers.pdf [file pone.0233468.s017.pdf]

Response to the reviewers

Reviewer #1: In this impressive study, Maes et al. found a novel role of ORY-2001 for brain memory function in various Alzheimer's disease animal models. Through a set of comprehensive in vitro and in vivo studies the authors have successfully proven the hypothesis that epigenetic modulation concerts neuronal plasticity, memory function, and behaviors. This paper is very strong scientifically. The authors have explored all the controls which prove the main theory. The amount of supporting data (including supplementary figures) is overwhelming. Last but not least, it is a nicely written and very well organized paper.

1. I have only a few minor comments about the effect of ORY-2001 for rescuing the memory deficit in SAMP8 mice. Generally, Morris water maze test is one of majorly well-known animal memory tests. In this regard, if the water maze test is added in figure 2 or 3, then the manuscript would be much stronger.

Indeed, the Morris Water Maze (MWM) is a standard test to measure memory deficits, and its use in SAMP8 mice has been described (Chen et al., 2004). We explored the MWM test in experiment 4. SAMR1 and SAMP8 groups (8 months old) learned to identify the location of the underwater platform during the training days. SAMP8 mice were slower than SAMR1 mice to reach the platform.

There was a tendency, but no significant difference for SAMR1 to spend more time in the platform quadrant than SAMP8 mice on the test day; i.e. the test did not provide an a priori good window for testing. SAMP8 mice behaved very poorly: their "total distance travelled" was much reduced compared to the SAMR1 control (a highly significant difference); and they exhibited "passive coping" (i.e. floating) behaviour rather than the normal swimming activity shown by the SAMR1 control strain. The lack of a significant difference between SAMR1 and SAMP8, together with the lack motivation for exploration and floating behaviour of SAMP8 mice invalidated this test as a means to evaluate memory in these animals.

On the contrary, SAMP8 and SAMR1 mice exhibited similar total exploration times in the NORT, allowing for a solid evaluation of the intended parameter. The differences in DI between SAMP8 and SAMR1 mice were highly significant and reproducible, which is why we opted for this particular test to explore different treatments, doses, duration, age, etc.

2. The conclusion part is not supportive enough and does not meet the level of this paper's data quality. Accordingly, I recommend that the authors could rewrite the conclusion part adequately.

We didn't want to be too redundant, but have now summarized the main conclusions in this section.

Anyways, despite the above critiques, I still believe this is a great paper that is worth being published in this journal.

Reviewer #2: The authors report the development of vafidemstat (ORY-2001), a brain penetrant inhibitor of KDM1A and MAOB. They show extensive in vitro and in vivo rodent data on its selectivity and effects on gene expression and behavior. They mine human neuropathology gene expression data to show that many of the target genes are increased in Alzheimer disease. Overall, they provide compelling data on the efficacy of this novel compound. However, the following items should be addressed:

1) Please rephrase lines 9-11 for clarity and define SAMP8 model.

Modified to:

ORY-2001 efficiently inhibits brain KDM1A at doses suitable for long term treatment, and corrects memory deficit as assessed in the novel object recognition testing the Senescence Accelerated Mouse Prone 8 (SAMP8) model for accelerated aging and Alzheimer's disease.

2) The authors state that ORY-2001 does not cause sedation but do not provide clear data to support this. They reference Fig 8A and S4 Fig C which do not appear to provide any measures of sedation.

Our statement on the absence of sedation by the compound derives from the previous evaluation of locomotor activity of these mice cohorts, as measured by the total distances travelled in open field test (Figure 7A), and also by assessment of the total number of entries in both arms of the EPM and rats (Figure 7C). Both parameters are standard measures of locomotor activity that are decreased after treatment with sedatives like Midazolam (Olson and Sherwin, 2006) or triazolam and lorazepam (Krsiak and Sulcova, 1990). Also, ORY-2001 did not have an anxiolytic effect in SAMP8 mice or in the rat isolation rearing model as measured by the time spent in the open arms of the EPM (Figure 7B and 7D). Therefore, we concluded there was no sedative effect. The lack of sedative effect is also exemplified in the S1 and S2 videos.

3) Lines 416 and 417 reference only the SAMP8 mice, yet it is stated that comparison to SAMR1 is made.

The phrase was modified to:

"We performed a genome-wide microarray-based survey on pooled PFC samples from *SAMR1 mice* and vehicle and *ORY-2001* treated SAMP8 mice from the "basal" cohort. All samples were compared to the vehicle treated SAMP8 mice, using two-color hybridization."

4) It is not clear what is meant by “regulated in LOAD vs normal samples” in line 485. This should be clarified.

Modified to “… are down-regulated in LOAD vs normal samples”. Some markers like S100A9 are clearly up-regulated, but we treated them earlier in the text. Many of the other makers identified are down-regulated, which could indeed reflect either a lack of response capacity or a loss of cells that normally express these genes, or both.

5) More details about the NCBI GEO: GSE44770 dataset should be provided including the numbers in each group, how groups were defined (clinically or pathologically), and age differences in order to best interpret this data. The possibility that differences between AD and control are due to neuronal loss/atrophy should be addressed.

The authors thank the reviewer for pointing this out, as indeed other potential variables should be considered as potential causes for differential gene expression between the “LOAD” and “control” groups. Age and gender are obvious ones to examine; and available in the public NCBI GEO: GSE44770 dataset. For better clarity, we have now included mean ages and numbers of the LOAD and AD groups in the figure legend.

According to the original article reporting the GSE44770 dataset, groups were defined both clinically and pathologically; subjects were diagnosed with LOAD at intake and each brain underwent extensive LOAD-related pathology examination.

We analysed the age of the subjects included in the study and we identified an important difference in mean age between the LOAD (N = 129; average age = 82.1 years) and control (N = 101; average age = 65.2 years) groups. We also reviewed the male/female ratio in the dataset and found that it was 4.3 in the control group and 0.9 in the LOAD group, a substantial difference. These differences could be a source for differential gene expression (although on the other hand it is well known that gender and age are important risk factors for AD).

To evaluate the relevance of the biomarkers with independence of age, we made a sub-selection of subjects, in a rather tight central age window (68 y < age < 75 y), in order to obtain LOAD and control subcohorts within the same age range (LOAD: N = 23; average age = 72.5 y; control: N = 21; average age = 71.4 y).

Of course, this drastic reduction of the size of the population reduces the power of the analysis, and the signal was lost for 3 genes (*CALB2*, *GNG4*, *NPAS4*) that showed smaller sized differences in mean gene expression in the total population. However, the rest of the genes remained highly significant: *S100A8* (FC = 4.24, p = 0.0002); *S100A9* (FC = 3.93, p = 0.0001); *EGR1* (FC = 0.54, p = 0.0022), *EGR*2 (FC = 0.57, p = 0.0028), *FOS* (FC = 1.71, p = 0.0044), *PENK1* (FC = 0.63, p = 0.0037), *RBM3* (FC = 0.43, p = 0.0004), *GAD1* (FC = 0.42, p < 0.0001), *DOC2A* (FC = 0.84, p = 0.0204), *UCHL1* (FC = 0.52, p = 0.0001). Such genes were confirmed to be differentially expressed, and all changes were in the same direction as the total sample population.

Subsequently, in order to evaluate the relevance of the biomarkers with independence of gender, we evaluated males and females separately. Almost all markers remained highly

significant and the direction and magnitude of change was similar in the female and male sub-cohorts, as can be seen in the images below (A - C).

A

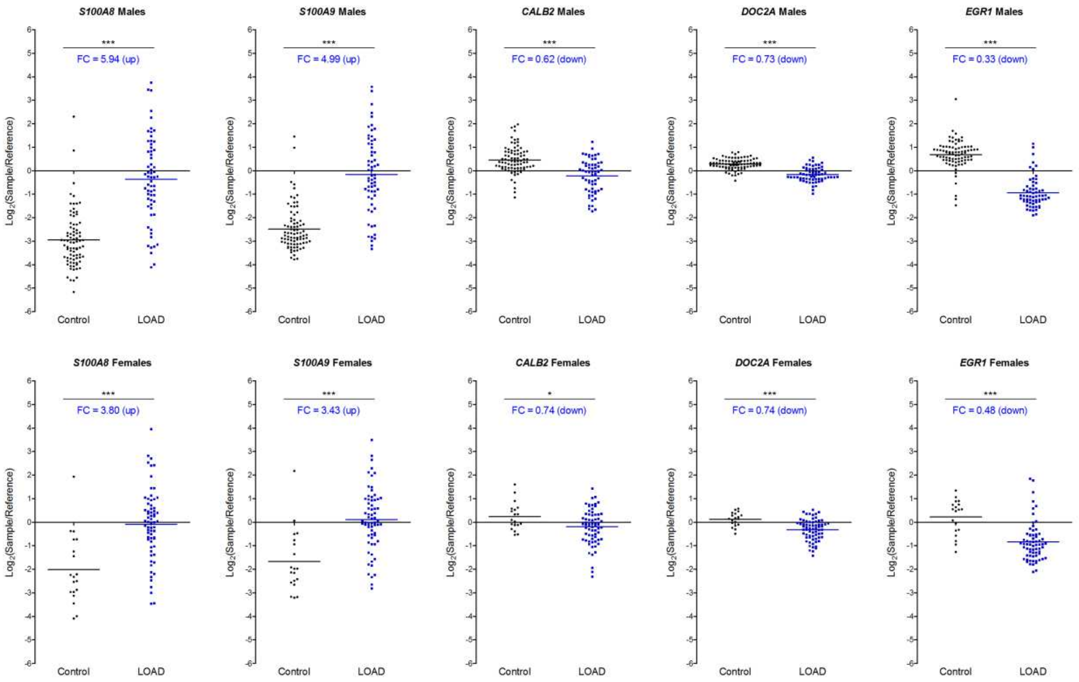
S100A8 Males
***
FC = 5.94 (up)
S100A9 Males
***
FC = 4.99 (up)
CALB2 Males
***
FC = 0.62 (down)
DOC2A Males
***
FC = 0.73 (down)
EGR1 Males
***
FC = 0.33 (down)
S100A8 Females
***
FC = 3.80 (up)
S100A9 Females
***
FC = 3.43 (up)
CALB2 Females
*
FC = 0.74 (down)
DOC2A Females
***
FC = 0.74 (down)
EGR1 Females
***
FC = 0.48 (down)
Log2(Sample/Reference)
Control
LOAD

B

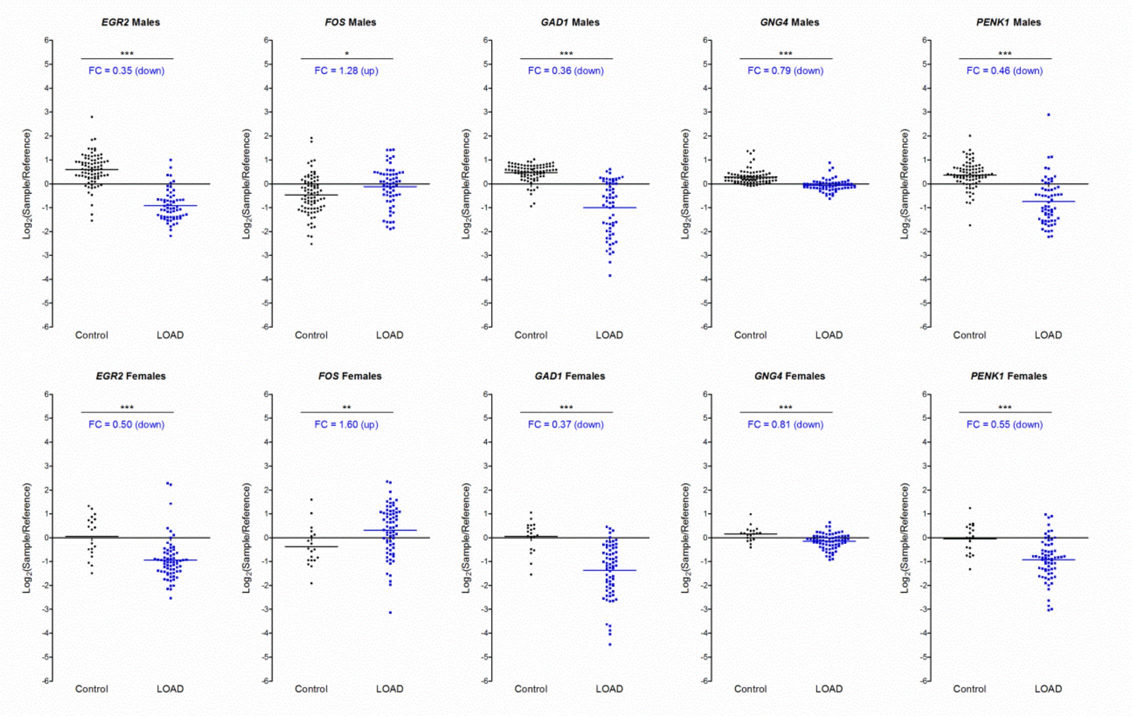
EGR2 Males
***
FC = 0.35 (down)
FOS Males
*
FC = 1.28 (up)
GAD1 Males
***
FC = 0.36 (down)
GNG4 Males
***
FC = 0.79 (down)
PENK1 Males
***
FC = 0.46 (down)
EGR2 Females
***
FC = 0.50 (down)
FOS Females
**
FC = 1.60 (up)
GAD1 Females
***
FC = 0.37 (down)
GNG4 Females
***
FC = 0.81 (down)
PENK1 Females
***
FC = 0.55 (down)
Log2(Sample/Reference)
Control
LOAD

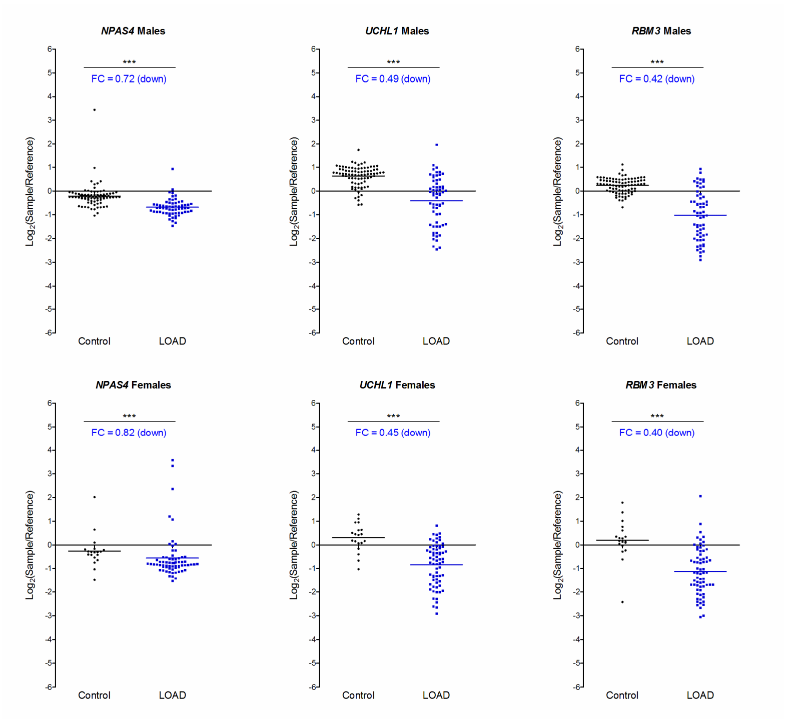

Therefore, unless the differences between LOAD and controls originate in yet another unidentified source of systematic variation, these genes appear to be robust markers reflecting genuine differences between LOAD and controls.

Finally, with respect to the possibility that differences between AD and control are due to neuronal loss / atrophy; we agree this is clearly a possibility for genes specifically expressed in neurons, like *UCHL1*.

6) For Figure 6C, images should be labelled with the relevant stain.
Corrected.

7) Interactor ELISA needs to be better defined and clearly delineated in the Figure 11 legend.

We have incorporated a schematic drawing of (chemoprobe) interactor ELISAs in Figure 11 and adapted the legend. For the interactor ELISAs, the antibodies combinations used for capture and detection are listed in the S2 File.
