# Supplementary material for: Modulation of KDM1A with vafidemstat rescues memory deficit and behavioral alterations
Source: PLoS One. 2020 May 29;15(5):e0233468. doi: 10.1371/journal.pone.0233468 (PMC7259601; doi:10.1371/journal.pone.0233468)
Supplement: Attachment — Submitted filename: Plos one review _1.26.2020-Dr Yoo.docx [file pone.0233468.s016.docx]

In this impressive study, Maes *et al*. found a novel role of ORY-2001 for brain memory function in various Alzheimer’s disease animal models. Through a set of comprehensive *in vitro* and *in vivo* studies the authors have successfully proven the hypothesis that epigenetic modulation concerts neuronal plasticity, memory function, and behaviors. This paper is very strong scientifically. The authors have explored all the controls which prove the main theory. The amount of supporting data (including supplementary figures) is overwhelming. Last but not least, it is a nicely written and very well organized paper.

1. I have only a few minor comments about the effect of ORY-2001 for rescuing the memory deficit in SAMP8 mice. Generally, Morris water maze test is one of majorly well-known animal memory tests. In this regard, if the water maze test is added in figure 2 or 3, then the manuscript would be much stronger.
2. The conclusion part is not supportive enough and does not meet the level of this paper’s data quality. Accordingly, I recommend that the authors could rewrite the conclusion part adequately.

Anyways, despite the above critiques, I still believe this is a great paper that is worth being published in this journal.

Junsang Yoo
